# Supplementary material for: Achieving Control of Asthma in Children in Africa (ACACIA): protocol of an observational study of children’s lung health in six sub-Saharan African countries
Source: BMJ Open. 2020 Mar 17;10(3):e035885. doi: 10.1136/bmjopen-2019-035885 (PMC7202730; doi:10.1136/bmjopen-2019-035885)
Supplement: Supplementary data [file bmjopen-2019-035885supp001.pdf]

Supplementary File
ACACIA Questionnaire - text only version

Participants will fill in a digital version of this questionnaire. They will be closely supported by facilitators throughout. Pictures are included in the digital questionnaire for all medication, including spacers, as well as cookers/main stoves, and light sources. In the digital version questions are automatically skipped, if the condition for the question is not fulfilled, or if they have answered the question at an earlier stage in the survey (e.g. as part of a conditional question).

Section 1: Personal details

1. **What is your name? ____**

2. **Date of birth (DD/MM/YYYY):** ***(digital: drop down menu)***

3. **Please find your home location on the map with the help of the visiting researcher.**

4. **Are you male or female?**

○ Male

○ Female

5. **Which school class or school year are you in? _____________**

6. **How would you describe your ethnicity?** ***(digital: drop down menu with follow up ' please specify for 'mixed race' and 'other')***

○ Black

○ White

○ East Asian, such as Chinese, Japanese, or Korean

○ South Asian, such as Indian, or Bangladeshi

○ Mixed race, please specify: _______________________

○ Other ethnicity, please specify: ___________________________

7. **Has a doctor, pharmacist, nurse, or healthcare worker ever said that you suffer from asthma?**

○ Yes

○ No

The questionnaire was based on work from the School-based Asthma Project, funded by the NIHR (National Institute for Health Research) CLAHRC (Collaboration for Leadership in Applied Health Research and Care) North Thames. The license for the Asthma Control Test (ACT) was donated to the ACACIA study through the NIHR CLAHRC North Thames as part of the initial work.

- Don't know

8. Has a doctor, pharmacist, nurse, or healthcare worker ever said that you suffer from any of the following conditions? (select all that apply)

- Atopic dermatitis, also sometimes known as eczema
- Hay fever and, or pollen allergy.
- Other allergies**, please specify:** ___________________________________
- Don't know

9. Have you been treated for Tuberculosis/TB?

- Yes
- No
- I'd rather not say
- Don't know

10. Does one or both of your parents suffer from any of the following conditions:

- Asthma
- Allergies
- Atopic dermatitis, also sometimes known as eczema
- None of the above
- Don't know

The questionnaire was based on work from the School-based Asthma Project, funded by the NIHR (National Institute for Health Research) CLAHRC (Collaboration for Leadership in Applied Health Research and Care) North Thames. The license for the Asthma Control Test (ACT) was donated to the ACACIA study through the NIHR CLAHRC North Thames as part of the initial work.

Section 2: Asthma Control Test (ACT) and GINA questions

ACT

1. **In the past 4 weeks, how much of the time did your asthma keep you from getting as much done at work, school or home?**

- ○ All of the time
- ○ Most of the time
- ○ Some of the time
- ○ A little of the time
- ○ None of the time

2. **In the past 4 weeks, how often have you had shortness of breath?**

- ○ More than once a day
- ○ Once a day
- ○ 3 to 6 times a week
- ○ Once or twice a week
- ○ Not at all

3. **In the past 4 weeks, how often did your asthma symptoms (wheezing, coughing, chest tightness, shortness of breath) wake you up at night or earlier than usual in the morning?**

- ○ 4 or more nights a week
- ○ 2 to 3 nights a week
- ○ Once a week
- ○ Once or twice (in 4 weeks)
- ○ Not at all

If you have a reliever or rescue inhaler

4. **In the past 4 weeks, how often have you used your rescue/reliever inhaler?**

- ○ 3 or more times per day
- ○ Once or twice per day
- ○ 2 or 3 times per week
- ○ Once a week or less
- ○ Not at all

The questionnaire was based on work from the School-based Asthma Project, funded by the NIHR (National Institute for Health Research) CLAHRC (Collaboration for Leadership in Applied Health Research and Care) North Thames. The license for the Asthma Control Test (ACT) was donated to the ACACIA study through the NIHR CLAHRC North Thames as part of the initial work.

5. How would you rate your asthma control during the last 4 weeks?

- Not controlled at all
- Poorly controlled
- Somewhat controlled
- Well controlled
- Completely controlled

GINA

1. In the past 4 weeks, have you had asthma symptoms during the day more than twice a week?

- Yes
- No

2. In the past 4 weeks, did you have any night waking due to asthma?

- Yes
- No

If you have a reliever or rescue inhaler

3. In the past 4 weeks, did you need your reliever more than twice/week?

- Yes
- No

4. In the past 4 weeks, did you have any activity limitation due to asthma?

- Yes
- No

The questionnaire was based on work from the School-based Asthma Project, funded by the NIHR (National Institute for Health Research) CLAHRC (Collaboration for Leadership in Applied Health Research and Care) North Thames. The license for the Asthma Control Test (ACT) was donated to the ACACIA study through the NIHR CLAHRC North Thames as part of the initial work.

Section 3: Medicines and adherence

1. **Do you take any medications for your asthma or wheeze?**

○ Yes

○ No

If yes,

2. **What type of medications do you usually use for your wheezing or asthma?** ***(pictures available in digital version)***

**Blue inhaler** Subumol, Salbutamol, Ventolin ☐	**Yellow inhaler** Albuterol, Proventil ☐	**Green inhaler** Salmeterol Serevent ☐	**Bricanyl** - Terbutaline DPI ☐	**Combivent HFA** Salbutamol Ipratropium ☐	**Duovent** Feneterol Ipratropium ☐	**Levosalbutamol Syrup** Levolin ☐

**Ventolin tablets** Salbutamol ☐	**Ascoril Syrup** ☐	**Salbutamol syrup** ☐	**Brown inhaler** Becasone, Beclometasone, Becotide ☐	**Red inhaler** Ciclesonide, Alvesco ☐	**Purple inhaler** Fluticasone/ Salmeterol Seretide ☐	**Red/white inhaler** Budesonide/ Formoterol Symbicort ☐

**Brown/white inhaler** Budesonide Pulmicort ☐	**Orange inhaler** Fluticasone Flixotide ☐	**Flixotide accuhaler DPI** ☐	**Seretide Accuhaler** ☐	**Asthmanex twisthaler** ☐	**Ciclovent, generic of Ciclesonide** ☐	**Steroid tablets**: Prednisolone ☐

**Theophylline tablets** or Nuelin SA ☐	**Ventolin tablets** Salbutamol ☐	**Franol, Asthmanol** Ephedrine theophylline ☐	**LTRA, Montelukast tablets** Topraz, Singulair, Sintrine, Monte-Air ☐	**Cough syrup** ☐	**Comple-mentary, herbal, or alternative medicines** ☐	**Nebulizer** ☐

☐ **I have other medicine,** Please name: ____

ACACIA questionnaire text only version, 28/01/2020 5

The questionnaire was based on work from the School-based Asthma Project, funded by the NIHR (National Institute for Health Research) CLAHRC (Collaboration for Leadership in Applied Health Research and Care) North Thames. The license for the Asthma Control Test (ACT) was donated to the ACACIA study through the NIHR CLAHRC North Thames as part of the initial work.

If you are using any MDI (puffer) inhaler:

3. Do you use a spacer for your inhaler at least some of the time

- Yes
- No
- Don't know

If you are using a spacer:

4. How often do you use your spacer?

- With each dose of medication
- Some of the time
- Never

If you are using a spacer:

5. Which type of spacer are you usually using?

- Home-made bottle spacer
- Bought plastic spacer

If you are taking reliever medicine when your symptoms get worse (for example blue or yellow reliever inhalers)

6. Do you sometimes <u>not</u> use your reliever medicine when you would need it? *(digital: look up button with list of reliever medicine)*

- All of the time
- Some of the time
- None of the time
- I do not have any reliever medicine

If you sometimes forget your reliever medicine,

7. **Would you like to tell us why?** ____

____

If you have regular preventer medicine, usually taken once or twice every day (for example a preventer inhaler)

8. Do you sometimes <u>not</u> take your regular daily preventer medicine? *(digital: look up button with list of preventer/controller medicine)*

- All of the time
- Some of the time
- None of the time
- I do not have any preventer medicine

The questionnaire was based on work from the School-based Asthma Project, funded by the NIHR (National Institute for Health Research) CLAHRC (Collaboration for Leadership in Applied Health Research and Care) North Thames. The license for the Asthma Control Test (ACT) was donated to the ACACIA study through the NIHR CLAHRC North Thames as part of the initial work.

If you sometimes forget to take your preventer medicine,

9. Would you like to tell us why?

____

____

The questionnaire was based on work from the School-based Asthma Project, funded by the NIHR (National Institute for Health Research) CLAHRC (Collaboration for Leadership in Applied Health Research and Care) North Thames. The license for the Asthma Control Test (ACT) was donated to the ACACIA study through the NIHR CLAHRC North Thames as part of the initial work.

Section 4 Medical attention

If you are taking medicine (section 3)

1. **How do you usually get your medicine for asthma or wheezing?**
   - Prescribed by a nurse, or a doctor at a clinic or hospital
   - Bought directly from a pharmacy without prescription
   - From a traditional healer or herbalist
   - I see different people about my medicines
   - My parent or carer gets the medicine for me, I usually don't come along
   - Other: ___________________________
   - Don't know

If prescribed by a nurse, or a doctor or bought from a pharmacist

2. **When you see your doctor, nurse or pharmacist about your medicine, would they usually ask you questions about your asthma or wheezing?**
   - Yes
   - Sometimes
   - They only talk to my parent or carer
   - No, they don't talk to me or my parents/carers about my asthma or wheezing
   - Don't know

3. **Where do you usually get medical help when your asthma or wheezing gets suddenly worse? Tick all that apply.**
   - Hospital
   - Emergency room
   - Local clinic or health centre
   - Pharmacy
   - Traditional healer, traditional doctor or herbalist
   - Other: ___________________________
   - Don't know

4. **In the past year, how often did you suddenly need to see a doctor or nurse at a hospital, emergency room, or clinic because of your asthma or wheezing?**
   - 4 or more times
   - 2-3 times
   - Once
   - Not at all
   - Don't know

The questionnaire was based on work from the School-based Asthma Project, funded by the NIHR (National Institute for Health Research) CLAHRC (Collaboration for Leadership in Applied Health Research and Care) North Thames. The license for the Asthma Control Test (ACT) was donated to the ACACIA study through the NIHR CLAHRC North Thames as part of the initial work.

5. In the past year, how many times would you have liked to get medical help because of your wheezing or asthma, but were not able to do so?

- ◯ 4 or more times
- ◯ 2-3 times
- ◯ Once
- ◯ Not at all
- ◯ Don't know

If at least once,
Would you like to tell us why? ____

The questionnaire was based on work from the School-based Asthma Project, funded by the NIHR (National Institute for Health Research) CLAHRC (Collaboration for Leadership in Applied Health Research and Care) North Thames. The license for the Asthma Control Test (ACT) was donated to the ACACIA study through the NIHR CLAHRC North Thames as part of the initial work.

Section 5: School activity

(if diagnosis of asthma)

1. How many times have you missed at least part of a school day due to your asthma in the past four weeks?

- 4 or more times
- 2-3 times
- Once
- Not at all

2. Do you feel that your asthma has a bad impact on how well you do in any of your classes or exams?

- My asthma doesn't have an impact at all
- My asthma has some impact
- My asthma has a big impact

3. Have you ever been teased, made fun of or bullied because of your asthma?

- A lot
- A little
- Never
- I'd rather not say

The questionnaire was based on work from the School-based Asthma Project, funded by the NIHR (National Institute for Health Research) CLAHRC (Collaboration for Leadership in Applied Health Research and Care) North Thames. The license for the Asthma Control Test (ACT) was donated to the ACACIA study through the NIHR CLAHRC North Thames as part of the initial work.

Section 6: Brief-Illness Perception (BIP) Questionnaire and peer support

(if diagnosis of asthma)

BIP

1. **How much does your asthma affect your life?**

0	1	2	3	4	5	6	7	8	9	10
No affect at all										Severely affects my life

2. **How long do you think your asthma will continue?**

0	1	2	3	4	5	6	7	8	9	10
A very short time										Forever

3. **How much control do you feel you have over your asthma?**

0	1	2	3	4	5	6	7	8	9	10
Absolutely no control										Extreme amount of control

4. **How much do you think your treatment can help your asthma?**

0	1	2	3	4	5	6	7	8	9	10
Not at all										Extremely helpful

5. **How much do you experience symptoms from your asthma?**

0	1	2	3	4	5	6	7	8	9	10
No symptoms at all										Many severe symptoms

6. **How concerned are you about your asthma?**

0	1	2	3	4	5	6	7	8	9	10
Not at all concerned										Extremely concerned

7. **How well do you feel you understand your asthma?**

0	1	2	3	4	5	6	7	8	9	10
Don't understand at all										Understand very clearly

8. **How much does your asthma affect you emotionally (e.g. does it make you angry, scared, upset or depressed)?**

0	1	2	3	4	5	6	7	8	9	10
Not at all affected emotionally										Extremely affected emotionally

The questionnaire was based on work from the School-based Asthma Project, funded by the NIHR (National Institute for Health Research) CLAHRC (Collaboration for Leadership in Applied Health Research and Care) North Thames. The license for the Asthma Control Test (ACT) was donated to the ACACIA study through the NIHR CLAHRC North Thames as part of the initial work.

PEER SUPPORT

1. **How important do you think peer support (from colleagues and friends) is for people with asthma?**

0	1	2	3	4	5	6	7	8	9	10
Not at all important										Extremely important

The questionnaire was based on work from the School-based Asthma Project, funded by the NIHR (National Institute for Health Research) CLAHRC (Collaboration for Leadership in Applied Health Research and Care) North Thames. The license for the Asthma Control Test (ACT) was donated to the ACACIA study through the NIHR CLAHRC North Thames as part of the initial work.

Section 7: Understanding about asthma

In this questionnaire we ask some questions about reliever and preventer inhalers. Ask the researchers with you to show you some examples.

**1. Your asthma would get worse if people smoke around you**	**Don't know**	**Agree**	**Disagree**
**2. Preventer inhalers only work if you feel them working immediately**	**Don't know**	**Agree**	**Disagree**
**3. Asthma is well controlled if you need to take a reliever inhaler once a day**	**Don't know**	**Agree**	**Disagree**
**4. Using a spacer with an asthma inhaler makes it easier to get the medicine into the lungs**	**Don't know**	**Agree**	**Disagree**
**5. A tight feeling in your chest can be a symptom for asthma**	**Don't know**	**Agree**	**Disagree**
**6. You can get addicted to asthma inhalers**	**Don't know**	**Agree**	**Disagree**
**7. Both stress and cold air can be triggers for asthma symptoms**	**Don't know**	**Agree**	**Disagree**
**8. People with asthma are less likely to get swollen airways**	**Don't know**	**Agree**	**Disagree**
**9. You should get a person straight to hospital if they cannot talk or walk anymore during an asthma attack**	**Don't know**	**Agree**	**Disagree**
**10. A spacer should only be used by small children**	**Don't know**	**Agree**	**Disagree**
**11. The reliever inhaler prevents the airways from becoming swollen**	**Don't know**	**Agree**	**Disagree**
**12. In an asthma emergency, you can give the reliever inhaler 10 puffs in a row**	**Don't know**	**Agree**	**Disagree**
**13. People with asthma should take a preventer inhaler when they have an asthma attack**	**Don't know**	**Agree**	**Disagree**
**14. Several young people die from asthma every year in my country**	**Don't know**	**Agree**	**Disagree**
**15. Young people sometimes make fun of asthma**	**Don't know**	**Agree**	**Disagree**

The questionnaire was based on work from the School-based Asthma Project, funded by the NIHR (National Institute for Health Research) CLAHRC (Collaboration for Leadership in Applied Health Research and Care) North Thames. The license for the Asthma Control Test (ACT) was donated to the ACACIA study through the NIHR CLAHRC North Thames as part of the initial work.

Section 8: Smoking, Home living, and Exposure

SMOKING

1. **Have you ever smoked cigarettes or tobacco?**
   - Yes
   - No
   - I'd rather not say

   If yes:

2. **How often do you smoke cigarettes or tobacco?**
   - Every day
   - 5-6 days a week
   - 3-4 days a week
   - 1-2 days a week
   - Less than once a week

3. **Does anyone smoke cigarettes or tobacco inside your home at least once a week?**
   - Yes
   - No
   - I'd rather not say

EXPOSURE

**4. Is the main cooker in your home usually used outside?**	**Don't know**	**Yes**	**No**
**5. Is the main cooker in your home electric?**	**Don't know**	**Yes**	**No**
**6. Does the road nearest your home have cars, buses, or trucks driving along it all the time?**	**Don't know**	**Yes**	**No**
**7. Do you have any animals or pets living with you at home?**	**Don't know**	**Yes**	**No**
**8. Do you live near a dump site where they burn waste at least once a month?**	**Don't know**	**Yes**	**No**
**9. Do you breathe in smoke from bush fires at least once a month?**	**Don't know**	**Yes**	**No**
**10. Does anyone living at your home use cleaning spray?**	**Don't know**	**Yes**	**No**
**11. Does anyone living at your home use insecticide spray?**	**Don't know**	**Yes**	**No**

The questionnaire was based on work from the School-based Asthma Project, funded by the NIHR (National Institute for Health Research) CLAHRC (Collaboration for Leadership in Applied Health Research and Care) North Thames. The license for the Asthma Control Test (ACT) was donated to the ACACIA study through the NIHR CLAHRC North Thames as part of the initial work.

**12. Does anyone living at your home use deodorant spray?**	**Don't know**	**Yes**	**No**
**13. Does anyone at your home burn mosquito coils?**	**Don't know**	**Yes**	**No**
**14. Does anyone at your home burn incense?**	**Don't know**	**Yes**	**No**
**15. Is your home sometimes air conditioned?**	**Don't know**	**Yes**	**No**
**16. Do you usually use electric lights at your house?**	**Don't know**	**Yes**	**No**

If the main cooker in your home IS NOT electric

17. What type of cooker is the main stove at your home? Tick all that apply.

☐	Gas cooker, or mixed gas and electric
☐	Open fire or three stone stove
☐	Cooker using coal, wood, or other solid fuel
☐	Cooker using kerosene, oil, or another liquid
☐	Solar cooker
☐	Others: ____
☐	Don't know

If you DO NOT usually use electric lights,

18. What sort of lighting do you use? Tick all that apply.

☐	**Candle (wax)**
☐	**Open fire**
☐	**Kerosene lamp**
☐	**Oil wick lamp**
☐	**Solar lantern**
☐	**Torch**
☐	**Rechargeable/ battery lamp**
☐	**Battery powered inverter**

The questionnaire was based on work from the School-based Asthma Project, funded by the NIHR (National Institute for Health Research) CLAHRC (Collaboration for Leadership in Applied Health Research and Care) North Thames. The license for the Asthma Control Test (ACT) was donated to the ACACIA study through the NIHR CLAHRC North Thames as part of the initial work.

☐	**Gasoline/diesel powered generator**
☐	**Crop residue**
☐	**Others: ____**
☐	**Don't know**

Researcher Section 9

to be filled in by the researcher

1. **Date of questionnaire session (DD/MM/YYYY): ______________________**

2. **School: ____**

3. **Section 1, Question 3: Lat:_____________, Long: ____________**

4. **What kind of area is the home in?**
   - o City or town
   - o Suburban
   - o Village or countryside

2. **Distance from home to nearest major road (map)**
   - o Less than 50m
   - o 50m-100m
   - o 100m-250m
   - o 250m-1000m
   - o More than 1000m

3. **Distance from school to nearest major road (measured on map)**
   - o Less than 50m
   - o 50m – 100m
   - o 100m - 250m
   - o 250m – 1000m
   - o More than 1000m

4. **School yard surface**
   - o Loose dirt
   - o Packed dirt
   - o Paved broken
   - o Paved

   Other: ____

The questionnaire was based on work from the School-based Asthma Project, funded by the NIHR (National Institute for Health Research) CLAHRC (Collaboration for Leadership in Applied Health Research and Care) North Thames. The license for the Asthma Control Test (ACT) was donated to the ACACIA study through the NIHR CLAHRC North Thames as part of the initial work.
