# Supplementary material for: Achieving Control of Asthma in Children in Africa (ACACIA): protocol of an observational study of children’s lung health in six sub-Saharan African countries
Source: BMJ Open. 2020 Mar 17;10(3):e035885. doi: 10.1136/bmjopen-2019-035885 (PMC7202730; doi:10.1136/bmjopen-2019-035885)
Supplement: Author's manuscript [file bmjopen-2019-035885.draft_revisions.pdf]

BMJ Open is committed to open peer review. As part of this commitment we make the peer review history of every article we publish publicly available.

When an article is published we post the peer reviewers' comments and the authors' responses online. We also post the versions of the paper that were used during peer review. These are the versions that the peer review comments apply to.

The versions of the paper that follow are the versions that were submitted during the peer review process. They are not the versions of record or the final published versions. They should not be cited or distributed as the published version of this manuscript.

BMJ Open is an open access journal and the full, final, typeset and author-corrected version of record of the manuscript is available on our site with no access controls, subscription charges or pay-per-view fees (http://bmjopen.bmj.com).

If you have any questions on BMJ Open's open peer review process please email info.bmjopen@bmj.com

BMJ Open

# Achieving Control of Asthma in Children In Africa (ACACIA). Protocol of an observational study of children's lung health in six sub-Saharan African countries.

| | |
|---|---|
| Journal: | *BMJ Open* |
| Manuscript ID | bmjopen-2019-035885 |
| Article Type: | Protocol |
| Date Submitted by the Author: | 20-Nov-2019 |
| Complete List of Authors: | Mosler, Gioia; Queen Mary University of London, Centre for Genomics and Child Health, Blizard Institute, Barts and The London School of Medicine and Dentistry<br>Oyenuga, Victoria; Queen Mary University of London, Centre for Genomics and Child Health, Blizard Institute, Barts and The London School of Medicine and Dentistry<br>Addo-Yobo, Emmanuel; Kwame Nkrumah University of Science and Technology, School of Medicine and Dentistry, College of Health Sciences<br>Adeyeye, Olayinka; Lagos State University College of Medicine<br>Masekela, R ; University of KwaZulu-Natal College of Health Sciences, Paediatrics and Child Health<br>Mujuru, Hilda; University of Zimbabwe College of Health Sciences<br>Nantanda, Rebecca; Makerere University, Lung Institute, Makerere College of Health Sciences<br>Rylance, Sarah; Wellcome Trust, Malawi-Liverpool -Wellcome (MLW) Trust Clinical Research Programme<br>Ticklay, Ismail; University of Zimbabwe College of Health Sciences<br>Grigg, Jonathan; Queen Mary University of London, Centre for Genomics and Child Health, Blizard Institute, Barts and The London School of Medicine and Dentistry |
| Keywords: | Asthma < THORACIC MEDICINE, Paediatric thoracic medicine < PAEDIATRICS, MEDICAL EDUCATION & TRAINING |
| | |

SCHOLARONE™
Manuscripts

*I, the Submitting Author has the right to grant and does grant on behalf of all authors of the Work (as defined in the below author licence), an exclusive licence and/or a non-exclusive licence for contributions from authors who are: i) UK Crown employees; ii) where BMJ has agreed a CC-BY licence shall apply, and/or iii) in accordance with the terms applicable for US Federal Government officers or employees acting as part of their official duties; on a worldwide, perpetual, irrevocable, royalty-free basis to BMJ Publishing Group Ltd ("BMJ") its licensees and where the relevant Journal is co-owned by BMJ to the co-owners of the Journal, to publish the Work in this journal and any other BMJ products and to exploit all rights, as set out in our licence.*

*The Submitting Author accepts and understands that any supply made under these terms is made by BMJ to the Submitting Author unless you are acting as an employee on behalf of your employer or a postgraduate student of an affiliated institution which is paying any applicable article publishing charge ("APC") for Open Access articles. Where the Submitting Author wishes to make the Work available on an Open Access basis (and intends to pay the relevant APC), the terms of reuse of such Open Access shall be governed by a Creative Commons licence – details of these licences and which Creative Commons licence will apply to this Work are set out in our licence referred to above.*

*Other than as permitted in any relevant BMJ Author's Self Archiving Policies, I confirm this Work has not been accepted for publication elsewhere, is not being considered for publication elsewhere and does not duplicate material already published. I confirm all authors consent to publication of this Work and authorise the granting of this licence.*

For peer review only

# Achieving Control of Asthma in Children In Africa (ACACIA). Protocol of an observational study of children's lung health in six sub-Saharan African countries.


## Authors:

Gioia Mosler[1]*, Victoria Oyenuga[1], Emmanuel Addo-Yobo[2], Olayinka Olufunke Adeyeye[3], Refiloe Masekela[4], Hilda Angela Mujuru[5], Rebecca Nantanda[6], Sarah Rylance[7], Ismail Ticklay[5], Jonathan Grigg[1]

**Affiliations:**

[1] Centre for Genomics and Child Health, Blizard Institute, Barts and The London School of Medicine and Dentistry, Queen Mary University of London, UK

[2] School of Medicine and Dentistry, College of Health Sciences, Kwame Nkrumah University of Science and Technology (KNUST), Ghana

[3] Lagos State University College of Medicine, Nigeria

[4] Nelson R Mandela School of Clinical Medicine, Department of Paediatrics and Child Health, University of KwaZulu Natal, South Africa

[5] University of Zimbabwe College of Health Sciences, Zimbabwe

[6] Makerere University Lung Institute, Makerere College of Health Sciences, Uganda

[7] Malawi-Liverpool -Wellcome (MLW) Trust Clinical Research Programme, Malawi

***Corresponding Author:**

Gioia Mosler

Address:

Queen Mary University of London
Blizard Institute
Centre for Genomics and Child Health
4 Newark St
London E1 2AT
UK

Telephone: +44 – (0)207 882 2361

Email: g.mosler@qmul.ac.uk


## Word Count

4000

For peer review only

# ABSTRACT

**Introduction**: Little is known about asthma control in the rising number of African children who suffer from this condition. The ACACIA study is an observational study collecting evidence about paediatric asthma in urban areas of Ghana, Malawi, Nigeria, South Africa, Uganda, and Zimbabwe. The primary objectives are 1) to identify 3000 children aged between 12 and 14 years with asthma symptoms; and 2) to assess their asthma control, current treatment, knowledge of and attitudes to asthma, and barriers to achieving good control. Secondary objective is to develop interventions addressing identified barriers to good symptom control.

**Methods and Analysis**: Each centre will undertake screening to identify 500 school children with asthma symptoms using questions from the Global Asthma Network's questionnaire. Children identified to have asthma symptoms will fill in a digital survey, including: Asthma Control Test (ACT), questions on medication usage and adherence, medical care, the Brief-Illness Perception questionnaire, and environmental factors. Exhaled nitric oxide (FeNO) testing, and pre and post bronchodilator spirometry will be performed. A subgroup of children will participate in focus group discussions. Results will be analysed using descriptive statistics and comparative analysis. Informed by these results, we will assess the feasibility of potential interventions, including the adaption of a UK-based theatre performance about asthma attitudes, and digital solutions to improve asthma management.

**Ethics and Dissemination**: The ACACIA study has been reviewed by the Queen Mary University of London (QMUL) Ethics of Research Committee in the UK. All African centres have received local ethical approval for this study. Study results will be published in academic journals, and at conferences. Study outputs will be communicated to the public via newsfeeds on the ACACIA website and twitter, and through news media outlets and other local dissemination.

**Registration:** The study has been registered and approved with ClinicalTrials.gov (record number: 269211).


# ARTICLE SUMMARY

## Strengths and limitations of this study

- The ACACIA study will record the number of children with asthma symptoms and proportion of these children with severe symptoms through school-based screening in Ghana, Malawi, Nigeria, South Africa, Uganda, and Zimbabwe.
- The ACACIA survey and focus group results will provide a wide range of information about barriers faced by children with asthma symptoms that prevent good asthma control and management.
- Although the study will recruit from six urban areas in sub-Saharan African countries, it can be readily expanded to other areas in other countries.
- ACACIA plans to test potential asthma management interventions with a special focus on asthma symptom control and hopes to obtain further funding for full intervention development in a second stage, which would address barriers to good asthma management identified through the study.

For peer review only

- One limitation is that children will be recruited in urban areas only, and previous studies have shown that asthma control levels in urban and rural areas are different with more severe asthma in urban compared to rural areas.

# INTRODUCTION

Until recently, childhood asthma in Africa was not considered to be a significant health issue. Surveys in African schools have found that the proportion of children aged 13 to 14 yr. with symptoms of asthma ranges between 10 to 20% in Central Africa, increasing to over 20% in South Africa. This high prevalence of asthma symptoms among children in Africa is thought to be partly due to the increase in urbanisation in Africa, which are linked to changes in lifestyle and environmental risk factors.(1) Uncontrolled asthma is associated with missed school days, repeated hospitalisation, risk of airway remodelling and general poor quality of life in the affected children.(2)

There have been few studies on childhood asthma is Africa, most of which focus on symptom prevalence.(1,3) A report from the Global Asthma Network (GAN) speculates that asthma control in African children is poor. Reasons for poor control are suspected not only to lie in the affordability and availability of asthma inhalers, but also in a lack of diagnosis, poorly coordinated asthma management, a lack of asthma knowledge in children, teachers and parents, and stigma associated with this long-term condition.(2) Some studies, have included aetiological research providing some indications about potential underlying factors of increased asthma prevalence, such as an association to air pollution.(2,4) Little is known however, about the impact asthma has on the life of young people and children in sub-Saharan African countries. One study by Iraqi et al. assessed influences of asthma control in 521 children across seven African countries of which Senegal, Niger, and Mali were located in sub-Saharan Africa. The study found that 45% of the children in the study, had sub-optimal asthma control according to the GINA (Global INitiative for Asthma) questions devised to assess asthma control. The study, furthermore, found that several factors were associated with poor control, including easy access to healthcare, and non-realisation of therapeutic education.(5) One study from Tanzania reports about fears of young people without asthma to play, sleep or eat with their peers who have asthma indicating an existing stigma about asthma.(6) There is a need for a comprehensive assessment of asthma control, asthma symptoms, and lung function in school children. The identification of possible facilitators and barriers for good symptom control will inform the design of relevant and scalable interventions.
The ACACIA study will draw on the methodology of the UK School-based Asthma Project (SAP). The SAP, found that 46% of London (UK) children and young people (age 12 – 18 yr.) had suboptimal asthma control. Further results from survey and focus group data revealed that many of them face a range of barriers to good asthma management, including lack of knowledge, forgetfulness and perceived stigma around asthma.(7) Based on this evidence, an intervention, called the "My Asthma in School Programme", was developed, which is currently running as a randomised control trial in London.(8) The ACACIA study will collect observational data about childhood asthma in a similar manner as the SAP in order to build the evidence-base for targeted and effective interventions in the future.

## Study Aims and Objectives

The ACACIA study aims to improve understanding of the burden that asthma imposes on school children, including the proportion of children with symptoms, symptom severity, asthma control, as well as deliver evidence of the highest quality on the barriers to achieving good asthma control that

For peer review only - http://bmjopen.bmj.com/site/about/guidelines.xhtml

contributes to poor asthma outcomes in urban African children. The primary objectives of this observational study are to i) identify a total of 3000 children between 12 and 14 years of age with asthma symptoms from Ghana, Malawi, Nigeria, South Africa, Uganda, and Zimbabwe; and ii) to assess their asthma control, lung function, current treatment and access to care, knowledge of and attitudes to asthma, as well as to identify the barriers to achieving good asthma control. The secondary objective is to explore ways to address the identified barriers, including the adaption of the 'In Control' theatre performance about understanding of asthma, from the SAP.

# METHODS AND ANALYSIS

The main study will follow a cross-sectional observational design. The study comprises of four stages:

Stage 1) Screening for asthma symptoms and asthma diagnosis in a 'Breathing Survey' using questions from the GAN questionnaire.(9) Participants, aged between 12 and 14 yr. in school will be recruited, as presented in the Participant Flow Chart (Figure 1). Children identified during screening with either wheezing symptoms in the previous 12 months, or those who report that they ever had asthma, or both will be invited to take part in the core data collection (Stage 2).

Stage 2) Core data collection includes the ACACIA questionnaire, pre and post bronchodilator spirometry and exhaled breath nitric oxide (FeNO) measurements.

Stage 3) Participants will be invited to take part in focus groups upon the conclusion of core data collection.

Stage 4) Potential interventions will be developed based on intermediate results of the data collection. One specific intervention, an educational theatre performance, will be piloted and tested for its effectiveness and impact on understanding asthma by young people.

Informed parental/guardian consent and child assent is collected in accordance with both country/local regulations and UK ethics regulations at three stages during data collection: 1) Screening Survey, 2) Core data collection, and 3) Focus groups (see also Figure 1) in five of the countries. In one country (Uganda) consent and assent for stages 1 and 2 are collected together.

For peer review only

## Preparing for data collection

To prepare for data collection, protocols and guidelines were developed centrally, as well as a digital platform for data entry. In the African study centres, research and fieldwork teams are recruited and trained. The training includes Spirometry and the measurement of Fractional Exhaled Nitric Oxide (FeNO). FeNO and Spirometry training is in accordance with American Thoracic Society (ATS) and European Respiratory Society (ERS) standards. Data entry and local storage systems are implemented with remote support from the central data management team.
All study materials, including information sheets and consent forms for parents, as well as assent and questionnaires for children are translated into local languages in areas where English is not universally used: Chichewa in Malawi (Blantyre), IsiZulu in South Africa (Durban), Luganda in Uganda (Makerere),

and Shona in Zimbabwe (Harare). Translations are using forwards and backwards translations. For the Asthma Control Test (ACT™, license donated to QMUL by GSK) translations are validated by the Mapi Research Trust.

# Recruitment

Each African study centre will recruit schools to the project until they have identified 500 children using a short screening questionnaire, called Breathing Survey. The target age for the central analysis is 12-14 yr. In some centres, school classes contain a wide age range of pupils as children progress through the school system at different rates. This is dependent on a number of factors, including academic ability, and periods of school absence. It was therefore decided that in those instances, centres can include older children of 15 and 16 yr. to improve ease of access to children within schools, and data may be used in local analyses. For the central analysis of the collected data, only children aged 12 to 14 yr. will be included. During school selection, researchers aim to reflect country-specific proportions of children educated in different school types (e.g. private schools and government-run schools), and aim to spread selected schools across the urban region in order to reflect geographical differences in socio-economic status. The number of schools recruited by each centre depends on the size of schools, as well as on asthma prevalence. For areas with low school attendance, additional recruitment of children attending acute care was considered. Subsequent feedback from local Principal Investigators confirmed however that only a small part of children would not attend school. Recruitment in schools was therefore considered to be sufficient.

# Screening for Asthma Symptoms

In the first stage of the study, researchers will visit school classes containing children of the target age group, provided consent and assent is given. Prior to completing the screening survey, informed consent and assent will be obtained in accordance with local and UK ethical review board requirements. Assistance with reading of the information sheet is provided to parents if necessary. The questions used for screening are taken from the validated GAN questionnaire to screen for asthma symptoms.(9)

# ACACIA survey and lung function tests

In a second stage, the adapted online survey will be delivered to children in schools by the research team who identified as having asthma symptoms or asthma in stage 1. In addition, children with asthma symptoms will undergo spirometry and FeNO measurement.
The ACACIA survey is adapted from a version of a UK-based SAP survey.(7) In schools where broadband is not available, the survey can be filled in offline, answers will be uploaded when connectivity is restored. The usage of electronic devices with long battery lives is encouraged in case of power cuts. Students who have difficulty using the digital survey will be supported by the research team.

The ACACIA survey includes:

1. Personal Details
2. Asthma Control Test (ACT™) and Global Initiative for Asthma (GINA) questions to assess asthma control
3. Medication and Adherence
4. Medical Attention and Access to Care
5. School Attendance and Attainment

For peer review only

6. Brief Illness Perception Questionnaire – Asthma
7. Asthma related Knowledge
8. Smoking, Home Living, and Exposure
9. Home Location and Distance to Nearest Major Road (researcher assisted)
10. School Specifications: Playground Surface, Distance to Next Major Road (researcher)

Several sections of the survey will be using questions successfully applied in the SAP survey(7), including a) medication adherence, b) unscheduled care, c) illness perception of asthma (measured using the Brief Illness Perception Questionnaire)(10), d) school absences, e) asthma knowledge, f) smoking and parental smoking. Additional questions are added about access to healthcare, and about environmental exposure were adapted from the IMPALA (International Multidisciplinary Programme to Address Lung health and TB in Africa) Question Set.(11)

Asthma control is one of the primary outcomes of this study and will be assessed using the Asthma Control Test (ACT)™(12), as well as GINA assessment of symptom control and future risk.(13) The ACT™ is a validated tool for assessing asthma control in people from the age of 12. The ACT consists of five items that assess activity limitation, shortness of breath, night time symptoms, use of reliever medication and overall rating of asthma control over the previous 4 weeks. The test score gives an indication as to whether the subject has controlled asthma or uncontrolled asthma. The GINA questions assess if a participant has well controlled, partly controlled or uncontrolled asthma symptoms with a set of four questions. Further insights into asthma symptoms will be provided by lung function data collected using Spirometry and FeNO testing.

After data has been collected at the school, children identified with asthma symptoms without a doctor-diagnosis of asthma, will receive a letter to the parents advising them to seek appropriate local healthcare or will be invited directly by local clinics where possible.

# Focus Groups

A subset of participants with asthma symptoms from each country will be taking part in focus groups after the core data collection to deepen the research team's understanding of possible facilitators and barriers to effective asthma management and to discuss possible components for a school-based asthma intervention. Additional focus groups will be held with parents and community groups. Informed consent and assent is collected for the focus group attendance in accordance with local and UK ethics regulations. Based on preliminary findings from the collected data, several focus group questions will be developed centrally for all study centres, each centre can then add topics of specific interest to their local context to the focus group discussions. There will be at least three focus groups with children and one adult focus group with parents/guardians and teachers. These focus groups will be held in each country. Focus group conversations will be recorded.

# Outcomes and Analysis

The primary outcome from this study will be the identification of the proportion of children with asthma symptoms and the proportion of those who have poor asthma symptom control in children aged 12 to 14 yr. in six sub-Saharan African countries. Control will primarily assessed by using:

- <u>Asthma Control Test</u>: using validated ACT™. The minimum score is 5 (poor control of asthma), the maximum score is 25 (complete control of asthma). An ACT score >19 indicates well-controlled asthma

Alternative ways of assessing asthma control will be explored, including: (14)

For peer review only

- Asthma Control according to GINA (Global INitiative for Asthma): GINA questionnaire (Global INitiative for Asthma) using four questions, assessing control of asthma symptoms. Outcome is 'well controlled' if none of the four questions is answered 'Yes', 'partly controlled' if one or two of the four questions is answered 'Yes', uncontrolled, if three or four of the four questions is answered 'Yes'.
- FeNO (fractional exhaled nitric oxide) measurement: A significant FeNO measurement will be deemed as a reading of ≥ 35 ppb, as per ATS/ERS guidelines for children.(15)
- Spirometry: Pre- and post-bronchodilator spirometry will be performed for all participants. Results and classified as obstructed if the $FEV_1$/FVC ratio is less than the Lower Limit of Normal. Obstructed spirometry will undergo a grading of severity based on the pre bronchodilator $FEV_1$ z-score. Post bronchodilator reversibility will be defined as an increase in the highest $FEV_1$ of ≥12% and 200ml from baseline.(16) The reference values used will be calculated from the global lung function (GLI) 2012 equations.(17)

  Spirometry parameters used for analysis include:
    - $FEV_1$, in litres, and expressed as percent predicted and z-score;
    - FVC, in litres, in percent predicted and z-score;
    - $FEV_1$/FVC ratio, FEV1/FVC LLN
    - FEF25-75 in litre/second and in percent predicted

Secondary outcomes:

- Percentage of children with asthma symptoms or asthma diagnosis, based on questions from the GAN (Global Asthma Network), screening tool.
- Assessment of barriers to good control:
    - Current treatment of asthma: set of questions about current medication, reported using descriptive statistics, and percentages
    - Adherence to medication: set of questions about adherence to medication, reported as descriptive statistics and percentages, as well as free text comments.
    - Access to medical care for asthma: set of questions related to access to medical care, reported using descriptive statistics, and percentages
    - Understanding of asthma: set of questions about asthma knowledge, maximum knowledge score is 13, minimum knowledge score is 0, and total range is 13. Higher values represent better knowledge.
    - Illness perception: the Brief Illness Perception questionnaire for asthma. Each of the 9 items in the Brief Illness Perception Questionnaire has a minimum score of 0 and a maximum score of 10. Overall score which represents the degree to which the illness is perceived as threatening or benign.(10,18)
- Asthma-related time off school: set of questions asking about asthma-related time off school, reported as descriptive statistics and percentages
- Assessment of environmental factors:
    - smoking: a set of questions assessing active and passive smoking, reported as descriptive statistics and percentages
    - Environmental factor assessment: a set of questions related to the environment of young people with asthma symptoms, reported using descriptive statistics, and percentages

For peer review only

Correlations with measures of asthma control to other variables, such as adherence to medication, and illness perception will be explored. Cross-analyses and comparison of the data between centres will also be undertaken. A detailed statistical analysis plan is being developed with the assistance of Statistician. Focus group discussions will be transcribed and keywords identified. Qualitative results from the focus groups will in addition inform the direction and acceptability of interventions aimed at removing barriers to poor asthma control.

# Intervention development

A preliminary analysis of the data from the survey and focus groups will inform ideas for possible interventions aimed at improving asthma outcomes and understanding around asthma in African children. One component of an intervention that will be assessed is a UK theatre intervention (play) adapted for an African context. The ACACIA study also aims to improve the digital resources available for asthma related services and for children who suffer from asthma. Digital solutions aimed at improved asthma self-management, such as smart peak flow meters, will be tested in the context of sub-Saharan African countries.

## Theatre intervention development and pilot

The original 'In Control' play was written by the Nigerian-born playwright Tunde Euba, and is currently running in London schools to secondary school year groups of up to 120 children by The Tramshed - Greenwich and Lewisham Young People's Theatre (GLYPT). This intervention is described in a Lancet perspective article.(19) The theatre will be adapted to the cultural context of sub-Saharan Africa by the original play writer in close collaboration with local arts and theatre groups. The adapted theatre intervention will be delivered to African children (aimed at 12 to 14 years of age) in a pilot study to at least five schools with two performances in each school, and an additional five performances to community groups at each African study centre. The best format for performances to community groups will be based on initial experiences with parents and the community during participant recruitment and data collection. Each centre is working with local theatre groups and arts departments to facilitate performances. Outcomes will be measured using short before and after questionnaires about knowledge and attitude towards asthma with additional questions on acceptability in the after-performance questionnaire. Results will be summarised using descriptive statistics and inform any potential future development of a full intervention, if further funding is provided.

# Public and Patient Involvement

Public and Patient Involvement (PPI) is highly encouraged within the project and training will be provided. Local and regional strategies for PPI are developed with reference to each local culture and build on existing links with external partners where feasible. During the set-up of the observational data collection, 15 children from three ACACIA countries between 11 and 15 yr. commented on the ACACIA questionnaire. Children are furthermore essential to the development and adaption of the theatre play through group discussions with the author held in two of the ACACIA countries.

For peer review only

# ETHICS AND DISSEMINATION

## Ethical review

The ACACIA study has been reviewed by the Queen Mary Ethics of Research Committee in the UK. Each collaborating African centre received ethical approval for the study locally with:

- Malawi: The College of Medicine Research Ethics Committee (COMREC), Reference No: P.10/18/2494, University of Malawi
- Nigeria: Lagos State /Lagos State University Teaching Hospital Health Research and Ethics Committee (NHREC04/04/2008), Reference No: LREC 06/10/1084
- Uganda: Mulago Hospital Research Ethics Committee (MHREC) number MHREC 1514; Institutional Review Board, and National Council for Science and Technology (UNCST) number SS 4940
- South Africa: Biomedical Research Ethics Committee of University of KwaZulu Natal BREC number: BF002/19 and Department of Basic Education KwaZulu Natal and Department of Health KwaZulu Natal
- Zimbabwe: The City Health Ethics Board, Joint Research Ethics Committee (JREC),the Medical Research Council of Zimbabwe (MRCZ); and the Research Council of Zimbabwe (RCZ)
- Ghana: Committee on Human Research, Publication and Ethics (CHRPE) - a joint committee of the School of Medical Science, Kwame Nkrumah University of Science and Technology (KNUST) and the Komfo Anokye Teaching Hospital (KATH), which is registered with the United States Department of Health and Human Services, Office for Human Research Protection (OHRP).

Study direction and progress will be guided by an international ACACIA steering group.

## Dissemination

Study results will be published in national and international peer review journals, and at conferences. Scientific results will be published as soon as it is reasonably practical and in compliance with the terms of the funding body. Publication will only be made after QMUL has received written permission from the funding body. Authorship will not necessarily be restricted to individuals named on this protocol; neither is authorship guaranteed to individual named on this protocol. Contributors who do not meet authorship criteria will be listed in ‘Acknowledgements’.
General study information will be displayed on the study’s website, as well as through its twitter account.

## Data Sharing, Access, and Release

The ACACIA study collects data from children in six African countries. Data will be collected by local research teams in each of the African centres. Participant’s identifiable data will be pseudo-anonymised before data is analysed or shared with the other centres through a digital platform system.

For peer review only

## Data protection and confidentiality:

Data collected from participants are stored locally in accordance with local and United Kingdom data protection legislations. The research team will be mindful to implement the highest data protection standards in all countries, especially where little or no local legislation exists. Access to identifiable data will only be possible within each country. The ACACIA central platform is hosted behind firewalls within a UK data storage facility. Storage and destruction of data are compliant with the UK Data Protection Act 2018. Only limited potentially identifiable data are contained within the central platform, specifically:

1. Age
2. Ethnicity
3. Gender
4. School (changed into an identifier)
5. Name (changed to an identifier)
6. Exact home location (changed into a square area (Mercator projection, reduced to 2 decimals, resulting in approximately 1km squares))

## Data monitoring

Intermittent random audit of data quality will be performed by members of each local investigating team. Audit by members from the co-ordinating centre in the UK will additionally be performed.

# ACKNOWLEDGEMENT

The authors would like to thank Professor Farida Fortune from Queen Mary University of London and her technological development team with Andy Carter, and Ian Sempill for their hard work towards developing innovative technological solutions for the study. Further thanks go to Jeremy James, Director of The Tramshed – GLYPT, who collaborates with the study to produce a tailored theatre performance around asthma knowledge and attitudes. The authors would furthermore like to thank all other members of the ACACIA steering group and Co-applicants to the grant for their valuable support of the study, including Professor Chris Griffiths, Professor Robert Walton, and Dr Liz Steed from Queen Mary University of London, as well as Professor Kevin Mortimer from the Liverpool School of Tropical Medicine, and Professor Mike Roberts from University College London Partners.

For peer review only

# REFERENCES


1. Adeloye D, Chan KY, Rudan I et al. An estimate of asthma prevalence in Africa: a systematic analysis. *Croat Med J.* 2013;54:519–31.
2. Global Asthma Network. The Global Asthma Report 2018. Auckland, New Zealand. 2018. http://globalasthmareport.org/Global Asthma Report 2018.pdf (accessed 29th October 2019).
3. Id OBO, Aderibigbe SA, Ayuk AC, Desalu OO, Oridota OE, Olufemi O, et al. The prevalence of asthma and allergic rhinitis in Nigeria: A nationwide survey among children, adolescents and adults. *Plos One* September 13 https://doi.org/10.1371/journal.pone.0222281 (accessed 29th October 2019).
4. Rylance S, Nightingale R, Naunje A et al. Lung health and exposure to air pollution in Malawian children (CAPS): a cross-sectional study. *Thorax* 2019;74:1070–7.
5. Iraqi B, Mahraoui C. Evaluation of asthma control among children in the Maghreb and sub-SaharIan Africa. *Eur Respir J.* 2016;48(suppl 60).
6. Shimwela M, Mwita JC, Mwandri M et al. Asthma prevalence, knownledge and perceptions among secondary school pupils in rural and urban coastal districts in Tanzania. *BMC Public Health* 2014;14:387.
7. Harris K, Mosler G, Williams SA, et al. Asthma control in London secondary school children. *J Asthma* 2017;54(10):1033–40.
8. Harris K, Mosler G, Grigg J. Intervention to improve adolescents' asthma control: a cluster randomised controlled trial protocol. *BMJ Open* 2019;9(4):e025867.
9. Global Asthma Network. Global Study Instruments for 13/14 year olds. http://www.globalasthmanetwork.org/surveillance/manual/study13.php (accessed 29th OCtober 2019).
10. Broadbent E, Petrie KJ, Main J et al. The Brief Illness Perception Questionnaire. *J Psychosom Res* 2006;60(6):631–7.
11. Liverpool School of Tropical Medicine. February 2018, IMPALA/ LuLi questionnaires https://www.lstmed.ac.uk/research/centres-and-units/impala/impala-outputs-resources (accessed 29th October 2019).
12. Schatz M, Sorkness CA, Li JT et al. Asthma Control Test: Reliability, validity, and responsiveness in patients not previously followed by asthma specialists. *J Allergy Clin Immunol* 2006;117(3):549–56.
13. Global Initiative for Asthma. Pocket Guide for Asthma Management and Prevention: GINA executive summary. 2018. 28 p.
14. Reddel HK, Taylor DR, Bateman ED et al. An official American Thoracic Society/European Respiratory Society statement: asthma control and exacerbations: standardizing endpoints for clinical asthma trials and clinical practice. *Am J Respir Crit Care Med* 2009 Jul 1;180(1):59-99.
15. Dweik RA, Boggs PB, Erzurum SC et al. An Official ATS Clinical Practice Guideline: Interpretation of Exhaled Nitric Oxide Levels (FENO) for Clinical Applications. *Am Thorac Soc Doc* 2011;184:602–15.
16. Swanney MP, Ruppel G, Enright PL et al. Using the lower limit of normal for the FEV 1/ FVC ratio reduces the misclassification of airway obstruction. *Thorax* 2008;63:1046–51.
17. Quanjer PH, Stanojevic S, Cole TJ et al. Multi-ethnic reference values for spirometry for the 3–95-yr age range: the global lung function 2012 equations. *Eur Respir J* 2012;40(6):1324–43.
18. Broadbent E, Wilkes C, Koschwanez H, et al. A systematic review and meta-analysis of the Brief Illness Perception Questionnaire. *Psychol Heal* 2015;30(11):1361–85.
19. Mosler G, Euba T. Taking control through drama. *Lancet* 2017;6736(17):1–2.


For peer review only

# AUTHORS CONTRIBUTIONS

GM is the main author of this manuscript and the Central Coordinator for ACACIA. JG is the Director of the ACACIA study providing overall direction and supervision. JG has contributed directly to this manuscript by proof-reading and commenting on its content. VO is the UK-based PhD student and Respiratory Physiologist of the study. She contributed to the manuscript through proof-reading and editing. All Principal Investigators (PIs) of each ACACIA centre (EA-Y, OA, RM, HAM, RN, SR, IT) have furthermore read and commented on the manuscript's content.

# FUNDING STATEMENT

This research was funded by the National Institute for Health Research (NIHR) (project reference) using UK aid from the UK Government to support global health research. The views expressed in this publication are those of the author(s) and not necessarily those of the NIHR or the UK Department of Health and Social Care (17/63/38).

# COMPETING INTEREST STATEMENTS

JG reports personal fees from GSK, personal fees from Vifor Pharmaceuticals, personal fees from Novartis, personal fees from BV Pharma, personal fees from AstraZeneca, outside the submitted work.

# LIST OF FIGURES

Figure 1: Participant Flow Chart

For peer review only

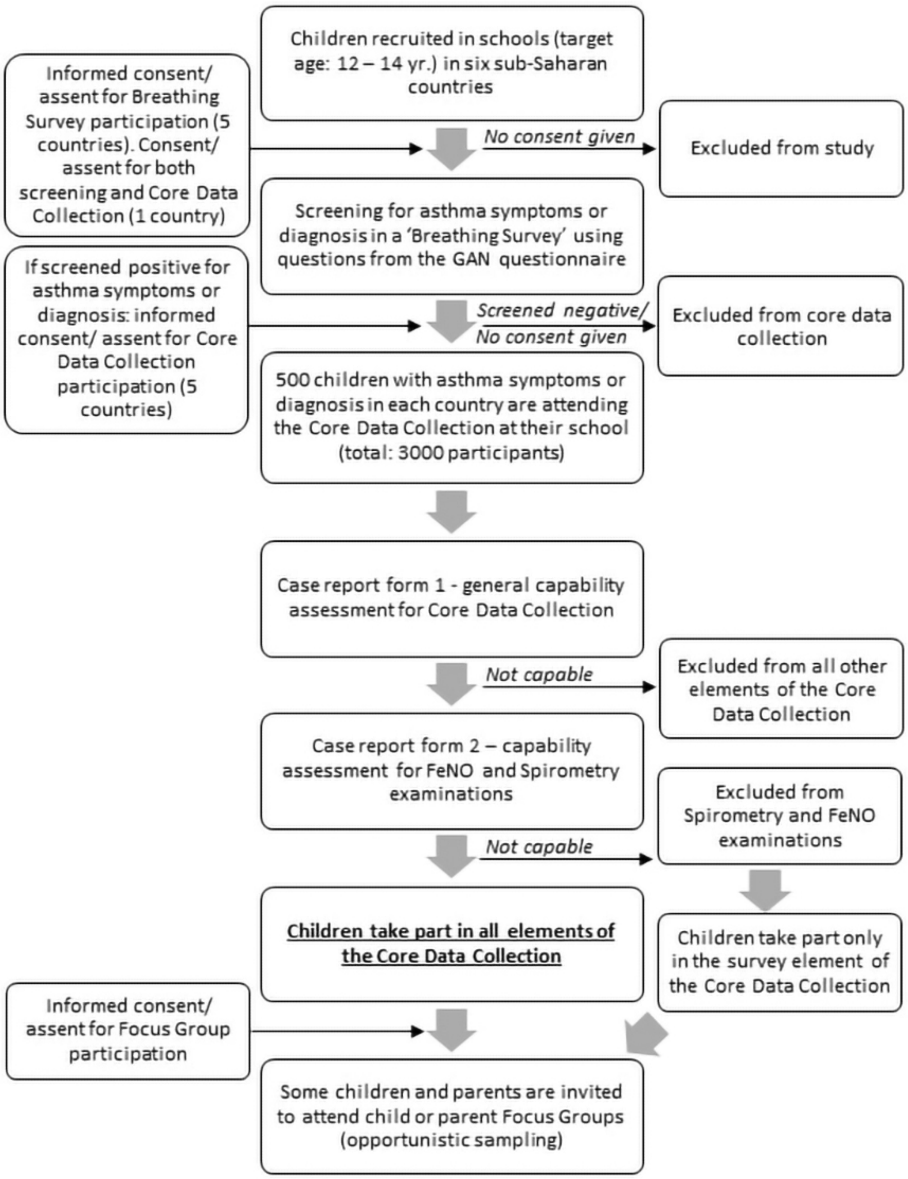


Participant Flow Chart

75x95mm (300 x 300 DPI)

BMJ Open

# Achieving Control of Asthma in Children In Africa (ACACIA). Protocol of an observational study of children's lung health in six sub-Saharan African countries.

| | |
|---|---|
| Journal: | *BMJ Open* |
| Manuscript ID | bmjopen-2019-035885.R1 |
| Article Type: | Protocol |
| Date Submitted by the Author: | 29-Jan-2020 |
| Complete List of Authors: | Mosler, Gioia; Queen Mary University of London, Centre for Genomics and Child Health, Blizard Institute, Barts and The London School of Medicine and Dentistry<br>Oyenuga, Victoria; Queen Mary University of London, Centre for Genomics and Child Health, Blizard Institute, Barts and The London School of Medicine and Dentistry<br>Addo-Yobo, Emmanuel; Kwame Nkrumah University of Science and Technology, School of Medicine and Dentistry, College of Health Sciences<br>Adeyeye, Olayinka; Lagos State University College of Medicine<br>Masekela, R ; University of KwaZulu-Natal College of Health Sciences, Paediatrics and Child Health<br>Mujuru, Hilda; University of Zimbabwe College of Health Sciences<br>Nantanda, Rebecca; Makerere University, Lung Institute, Makerere College of Health Sciences<br>Rylance, Sarah; Wellcome Trust, Malawi-Liverpool -Wellcome (MLW) Trust Clinical Research Programme<br>Ticklay, Ismail; University of Zimbabwe College of Health Sciences<br>Grigg, Jonathan; Queen Mary University of London, Centre for Genomics and Child Health, Blizard Institute, Barts and The London School of Medicine and Dentistry |
| <b>Primary Subject Heading</b>: | Respiratory medicine |
| Secondary Subject Heading: | Global health, Paediatrics, Public health |
| Keywords: | Asthma < THORACIC MEDICINE, Paediatric thoracic medicine < PAEDIATRICS, MEDICAL EDUCATION & TRAINING |
| | |

SCHOLARONE™
Manuscripts

*I, the Submitting Author has the right to grant and does grant on behalf of all authors of the Work (as defined in the below author licence), an exclusive licence and/or a non-exclusive licence for contributions from authors who are: i) UK Crown employees; ii) where BMJ has agreed a CC-BY licence shall apply, and/or iii) in accordance with the terms applicable for US Federal Government officers or employees acting as part of their official duties; on a worldwide, perpetual, irrevocable, royalty-free basis to BMJ Publishing Group Ltd ("BMJ") its licensees and where the relevant Journal is co-owned by BMJ to the co-owners of the Journal, to publish the Work in this journal and any other BMJ products and to exploit all rights, as set out in our licence.*

*The Submitting Author accepts and understands that any supply made under these terms is made by BMJ to the Submitting Author unless you are acting as an employee on behalf of your employer or a postgraduate student of an affiliated institution which is paying any applicable article publishing charge ("APC") for Open Access articles. Where the Submitting Author wishes to make the Work available on an Open Access basis (and intends to pay the relevant APC), the terms of reuse of such Open Access shall be governed by a Creative Commons licence – details of these licences and which Creative Commons licence will apply to this Work are set out in our licence referred to above.*

*Other than as permitted in any relevant BMJ Author's Self Archiving Policies, I confirm this Work has not been accepted for publication elsewhere, is not being considered for publication elsewhere and does not duplicate material already published. I confirm all authors consent to publication of this Work and authorise the granting of this licence.*

For peer review only

# Achieving Control of Asthma in Children In Africa (ACACIA). Protocol of an observational study of children's lung health in six sub-Saharan African countries.


## Authors:

Gioia Mosler[1]*, Victoria Oyenuga[1], Emmanuel Addo-Yobo[2], Olayinka Olufunke Adeyeye[3], Refiloe Masekela[4], Hilda Angela Mujuru[5], Rebecca Nantanda[6], Sarah Rylance[7], Ismail Ticklay[5], Jonathan Grigg[1]

**Affiliations:**

[1] Centre for Genomics and Child Health, Blizard Institute, Barts and The London School of Medicine and Dentistry, Queen Mary University of London, UK

[2] School of Medicine and Dentistry, College of Health Sciences, Kwame Nkrumah University of Science and Technology (KNUST), Ghana

[3] Lagos State University College of Medicine, Nigeria

[4] Nelson R Mandela School of Clinical Medicine, Department of Paediatrics and Child Health, University of KwaZulu Natal, South Africa

[5] University of Zimbabwe College of Health Sciences, Zimbabwe

[6] Makerere University Lung Institute, Makerere College of Health Sciences, Uganda

[7] Malawi-Liverpool -Wellcome (MLW) Trust Clinical Research Programme, Malawi

***Corresponding Author:**

Gioia Mosler

Address:

Queen Mary University of London
Blizard Institute
Centre for Genomics and Child Health
4 Newark St
London E1 2AT
UK

Telephone: +44 – (0)207 882 2361

Email: g.mosler@qmul.ac.uk


## Word Count

4644

For peer review only

# ABSTRACT

**Introduction**: Little is known about asthma control in the rising number of African children who suffer from this condition. The ACACIA study is an observational study collecting evidence about paediatric asthma in urban areas of Ghana, Malawi, Nigeria, South Africa, Uganda, and Zimbabwe. The primary objectives are 1) to identify 3000 children aged between 12 and 14 years with asthma symptoms; and 2) to assess their asthma control, current treatment, knowledge of and attitudes to asthma, and barriers to achieving good control. Secondary objective is to develop interventions addressing identified barriers to good symptom control.

**Methods and Analysis**: Each centre will undertake screening to identify 500 school children with asthma symptoms using questions from the Global Asthma Network's questionnaire. Children identified to have asthma symptoms will fill in a digital survey, including: Asthma Control Test (ACT), questions on medication usage and adherence, medical care, the Brief-Illness Perception questionnaire, and environmental factors. Exhaled nitric oxide (FeNO) testing, and pre and post bronchodilator spirometry will be performed. A subgroup of children will participate in focus group discussions. Results will be analysed using descriptive statistics and comparative analysis. Informed by these results, we will assess the feasibility of potential interventions, including the adaption of a UK-based theatre performance about asthma attitudes, and digital solutions to improve asthma management.

**Ethics and Dissemination**: The ACACIA study has been reviewed by the Queen Mary University of London (QMUL) Ethics of Research Committee in the UK. All African centres have received local ethical approval for this study. Study results will be published in academic journals, and at conferences. Study outputs will be communicated to the public via newsfeeds on the ACACIA website and twitter, and through news media outlets and other local dissemination.

**Registration:** The study has been registered and approved with ClinicalTrials.gov (record number: 269211).

# ARTICLE SUMMARY

## Strengths and limitations of this study

- The ACACIA study will record the number of children with asthma symptoms and proportion of these children with severe symptoms through school-based screening in Ghana, Malawi, Nigeria, South Africa, Uganda, and Zimbabwe.
- The ACACIA survey and focus group results will provide a wide range of information about barriers faced by children with asthma symptoms that prevent good asthma control and management.
- Although the study will recruit from six urban areas in sub-Saharan African countries, it can be readily expanded to other areas in other countries.
- ACACIA plans to test potential asthma management interventions with a special focus on asthma symptom control and hopes to obtain further funding for full intervention development in a second stage, which would address barriers to good asthma management identified through the study.

For peer review only

- One limitation is that children will be recruited in urban areas only, and previous studies have shown that asthma control levels in urban and rural areas are different with more severe asthma in urban compared to rural areas.

# INTRODUCTION

Until recently, childhood asthma in Africa was not considered to be a significant health issue. Previous surveys of African children (age 13 - 14 yr.) in schools have found that the proportion of children with symptoms of asthma ranges between 10 to 20% in Central Africa, increasing to over 20% in South Africa. This high prevalence of asthma symptoms among children in Africa is thought to be partly due to the increase in urbanisation in Africa, which are linked to changes in lifestyle and environmental risk factors.(1) Uncontrolled asthma is associated with missed school days, repeated hospitalisation, risk of airway remodelling and general poor quality of life in the affected children.(2)

There have been few studies on childhood asthma in Africa, most of which focus on symptom prevalence or associations of asthma prevalence with allergies or air pollution.(1,2,3,4) Little is known however, about the impact asthma has on the life of young people and children in sub-Saharan African countries. A report from the Global Asthma Network (GAN) speculates that asthma control in African children is poor. Reasons for poor control are suspected not only to lie in the affordability and availability of asthma inhalers, but also in a lack of diagnosis, poorly coordinated asthma management, a lack of asthma knowledge in children, teachers and parents, and stigma associated with this long-term condition.(2) One study by Iraqi et al. assessed influences of asthma control in 521 children across seven African countries of which Senegal, Niger, and Mali were located in sub-Saharan Africa. The study found that 45% of the children in the study, had sub-optimal asthma control according to the GINA (Global INitiative for Asthma) questions devised to assesses asthma control. The study, furthermore, found that several factors were associated with poor control, including easy access to healthcare, and failure to carry out patient education.(5) One study from Tanzania reports about fears of young people without asthma to play, sleep or eat with their peers who have asthma indicating an existing stigma about asthma.(6) There is a need for a comprehensive assessment of asthma control, asthma symptoms, and lung function in school children. The identification of possible facilitators and barriers for good symptom control will inform the design of relevant and scalable interventions.

The ACACIA study will draw on the methodology of the UK School-based Asthma Project (SAP). The SAP, found that 46% of 766 London (UK) children and young people (age 12 – 18 yr.) had suboptimal asthma control. Further results from survey and focus group data revealed that many of them face a range of barriers to good asthma management, including lack of knowledge, forgetfulness and perceived stigma around asthma.(7) Based on this evidence, an intervention, called the "My Asthma in School Programme", was developed, comprising of two components: A) a theatre workshop for all children in school year groups 7 and 8, and B) a self-management workshops for children with asthma. A theory-based cluster randomised controlled trial is currently running in London to test this intervention with 3, 6, and 12 month follow-up.(8)

The ACACIA study will collect observational data about childhood asthma in a similar manner as the SAP in order to build the evidence-base for targeted and effective interventions in the future. ACACIA will collect data related to asthma from 12 to 14 year old adolescents across sub-Saharan Africa. The study wants to understand asthma during adolescence, when there is a shift from parental to personal symptom management, and when optimal self-management standards of asthma could be introduced in a future intervention. ACACIA will furthermore focus on urban areas in sub-Saharan Africa, as prevalence of childhood asthma is particularly high in urban areas of the region.(1)

## Study Aims and Objectives

The ACACIA study aims to improve understanding of the burden that asthma imposes on school children, including the proportion of children with symptoms, symptom severity, and asthma control. A secondary aim of the study is to deliver evidence of the highest quality on the barriers to achieving good asthma control that contributes to poor asthma outcomes in urban African children. The primary objectives of this observational study are to i) identify a total of 3000 children between 12 and 14 years of age with asthma symptoms from Ghana, Malawi, Nigeria, South Africa, Uganda, and Zimbabwe; and ii) to assess their asthma control, lung function, current treatment and access to care, knowledge of and attitudes to asthma, as well as to identify the barriers to achieving good asthma control. The secondary objective is to explore ways to address the identified barriers, including the adaption of the 'In Control' theatre performance about understanding of asthma, from the SAP.

# METHODS AND ANALYSIS

The main study will follow a cross-sectional observational design. The study comprises of four stages:

Stage 1) Screening for asthma symptoms and asthma diagnosis in a Breathing Survey in school using questions from the GAN questionnaire.(9) Participants, aged between 12 and 14 yr. in school will be recruited, as presented in the Participant Flow Chart (Figure 1). Children identified during screening with either wheezing symptoms in the previous 12 months, or those who report that they ever had asthma, or both will be invited to take part in the core data collection (Stage 2).

Stage 2) Core data collection at school includes the ACACIA questionnaire, pre and post bronchodilator spirometry and exhaled breath nitric oxide (FeNO) measurements.

Stage 3) Participants with symptoms of asthma will be invited to take part in focus group discussions upon the conclusion of core data collection.

Stage 4) Potential interventions will be developed based on intermediate results of the data collection. One specific intervention, an educational theatre performance, will be adapted from a UK-based play about asthma, called 'In Control, piloted and tested for its effectiveness and impact on understanding asthma by young people in Africa.

Informed parental/guardian consent and child assent is collected in accordance with both country/local regulations and UK ethics regulations at three stages during data collection: 1) Screening Survey, 2) Core data collection, and 3) Focus groups (see also Figure 1) in five of the countries. In one country (Uganda) consent and assent for stages 1 and 2 are collected together, as this was the preferred design in regards to the country's ethical approval.

For peer review only

The study will be conducted simultaneously in schools of six African urban areas: Kumasi - Ghana; Blantyre - Malawi; Lagos – Nigeria; Durban - South Africa; Makerere – Uganda; and Harare – Zimbabwe. Work in each of these urban areas is led by a principle investigator at a local university and conducted by their team of researchers and fieldworkers.

# Preparing for data collection

To prepare for data collection, protocols and guidelines were developed centrally, as well as a digital platform for data entry. The digital platform comprises of a central research database, hosted by Microsoft's Azure Platform in the UK, which receives and stores the anonymised data collected by all field teams, and local databases in each centre, stored on servers within the university, which allow storage of personal data. A suite of bespoke software enables for data transfer and reporting. In the African study centres, research and fieldwork teams are recruited and trained. Field workers will be trained to perform quality assured spirometry using the NDD Easy On-PC and the use of the NIOX VERO to measure Fractional Exhaled Nitric Oxide (FeNO). FeNO and Spirometry training is in accordance with American Thoracic Society (ATS) and European Respiratory Society (ERS) standards, 2005. Testing will be carried out by the field workers, who have undergone training, using the NDD Easy On-PC and the NIOX VERO. Standard Operating procedures for Spirometry and FeNO testing will also be provided to ensure standardisation and quality. Data entry and local storage systems are implemented with remote support from the central data management team.
All study materials, including information sheets and consent forms for parents, as well as assent and questionnaires for children are translated into local languages in areas where English is not universally used: Chichewa in Malawi (Blantyre), IsiZulu in South Africa (Durban), Luganda in Uganda (Makerere), and Shona in Zimbabwe (Harare). Translations are using forwards and backwards translations. For the Asthma Control Test (ACT™, license donated to QMUL by GSK) translations are validated by the Mapi Research Trust.

# Recruitment

Each African study centre will recruit schools to the project until they have identified 500 children using a short screening questionnaire, called Breathing Survey. Recruitment of schools to the project started in July 2019 and school-based data collection is planned to finish before January 2021. The target age for the central analysis is 12-14 yr. In some centres, school classes contain a wide age range of pupils as children progress through the school system at different rates, dependent on a number of factors, including academic ability, and periods of school absence. It was therefore decided that, centres can include older children of 15 and 16 yr. to improve ease of access to children within schools. This additionally collected data will be excluded from the planned central analysis. Local research centres may decide to use the data in an analysis at a later stage. During school selection, researchers aim to reflect country-specific proportions of children educated in different school types (e.g. private schools and government-run schools), and aim to spread selected schools across the urban region in order to reflect geographical differences in socio-economic status. The number of schools recruited by each centre depends on the size of schools, as well as on asthma prevalence. For areas with low school attendance, additional recruitment of children attending acute care was considered. Subsequent feedback from local Principal Investigators confirmed however that only a small part of children would not attend school. Recruitment in schools was therefore considered to be sufficient.

For peer review only - http://bmjopen.bmj.com/site/about/guidelines.xhtml

# Screening for Asthma Symptoms

Prior to completing the screening survey, informed consent and assent will be obtained in accordance with local and UK ethical review board requirements. The research teams are working closely with the schools to obtain consent from the parents ahead of data collection. Information sheets about the study are provided to parents and children, outlining the research, the participant's involvement, and important aspects of participation, such as the right to withdraw. The local research teams are available for any further questions and for assistance with reading of the information sheet, if necessary. Researchers will visit school classes containing children of the target age group to facilitate the filling in of the Breathing Survey, provided consent and assent is given. The questions used in the Breathing Survey are taken from the validated GAN questionnaire to screen for asthma symptoms, using its demographic questions, and questions 2 – 7 about breathing.(9)

# ACACIA survey and lung function tests

In a second stage, the adapted online survey will be delivered to children in schools by the research team who identified as having asthma symptoms or asthma diagnosis in stage 1. Following existing studies the percentage of children with asthma symptoms is expected to be between 10% and 20%.(1) In addition, children with asthma symptoms will undergo spirometry and FeNO measurement.
The ACACIA survey is adapted from a version of a UK-based SAP survey,(7) a non-digital list of the survey questions can be found in the online supplementary file. In schools where broadband is not available, the survey can be filled in offline, answers will be uploaded when connectivity is restored. The usage of electronic devices with long battery lives is encouraged in case of power cuts. Students who have difficulty using the digital survey will be supported by the research team.

The ACACIA survey includes:

1. Personal Details
2. Asthma Control Test (ACT™) and Global Initiative for Asthma (GINA) questions to assess asthma control
3. Medication and Adherence
4. Medical Attention and Access to Care
5. School Attendance and Attainment
6. Brief Illness Perception Questionnaire – Asthma
7. Asthma related Knowledge
8. Smoking, Home Living, and Exposure
9. Home Location and Distance to Nearest Major Road (researcher assisted)
10. School Specifications: Playground Surface, Distance to Next Major Road (researcher)

Several sections of the survey will be using questions successfully applied in the SAP survey(7), including a) medication adherence, b) unscheduled care, c) illness perception of asthma (measured using the Brief Illness Perception Questionnaire)(10), d) school absences, e) asthma knowledge, f) smoking and parental smoking. Additional questions about access to healthcare, and about environmental exposure were adapted from the set of IMPALA (International Multidisciplinary Programme to Address Lung health and TB in Africa) questionnaires.(11)
Asthma control is one of the primary outcomes of this study and will be assessed using the Asthma Control Test (ACT)™(12), as well as GINA assessment of symptom control and future risk.(13) The ACT™ is a validated tool for assessing asthma control in people from the age of 12. The ACT consists of five

For peer review only

items that assess activity limitation, shortness of breath, night time symptoms, use of reliever medication and overall rating of asthma control over the previous 4 weeks. The test score gives an indication as to whether the subject has controlled asthma or uncontrolled asthma. GINA uses four questions about symptoms, reliever usage, and activity limitations to assess if a participant has well controlled, partly controlled or uncontrolled asthma symptoms with a set of four questions.
Further insights into asthma symptoms will be provided by lung function data collected using Spirometry and FeNO testing. Case report forms will be used to gather information on any medications a participant is taking and the presence of any absolute or relative contraindications to FeNO or Spirometry measurement. Calibration verification (Linearity check) will be performed every day, prior to testing, at each data collection location. Pre- and post-bronchodilator spirometry will be performed for all participants who have no contraindications. 400 µg Salbutamol will be given, post baseline spirometry, via a pMDI and spacer. Spirometry quality will then be reviewed and agreed by two spirometry specialists before deemed acceptable for use in analysis. The reference values used will be calculated from the global lung function (GLI) 2012 equations. (14)
After data has been collected at the school, children identified with asthma symptoms without a doctor-diagnosis of asthma, will receive a letter to the parents advising them to seek appropriate local healthcare or will be invited directly by local clinics where possible.

# Focus Groups

After the core data collection, focus groups will be held, to deepen the research team's understanding of possible facilitators and barriers to effective asthma management and to discuss possible components for a school-based asthma intervention. At each centre, ACACIA participants from at least three schools are selected to take part in 1-hour long focus group discussions with five to eight participants. In each school there will be a) one discussion with children who have an asthma diagnosis, and b) one discussion with children who have symptoms of asthma, but no diagnosis. An additional six focus groups per centre will be held with parents and teachers. Informed consent and assent is collected for the focus group attendance in accordance with local and UK ethics regulations. Based on preliminary findings from the collected questionnaire data, several focus group questions will be developed for all study centres, each centre can then add topics of specific interest to their local context to the focus group discussions. These focus groups will be held by trained local researchers in each country. Focus group conversations will be recorded. A detailed protocol for the facilitation and analysis of focus groups will be developed for all centres.

# Outcomes and Analysis

The primary outcome from this study will be the identification of the proportion of children with asthma symptoms and the proportion of those who have poor asthma symptom control in children aged 12 to 14 yr. in six sub-Saharan African countries. Control will primarily assessed by using:

- Percentage of children with asthma symptoms or asthma diagnosis, based on questions from the GAN (Global Asthma Network), screening tool.
- Asthma Control Test: using validated ACT™. The minimum score is 5 (poor control of asthma), the maximum score is 25 (complete control of asthma). An ACT score >19 indicates well-controlled asthma

Alternative ways of assessing asthma control will be explored, including: (15)

- Asthma Control according to GINA (Global INitiative for Asthma): GINA questionnaire (Global INitiative for Asthma) using four questions, assessing control of asthma symptoms. Outcome is

For peer review only - http://bmjopen.bmj.com/site/about/guidelines.xhtml

'well controlled' if none of the four questions is answered 'Yes', 'partly controlled' if one or two of the four questions is answered 'Yes', uncontrolled, if three or four of the four questions is answered 'Yes'.

- FeNO (fractional exhaled nitric oxide) measurement: A significant FeNO measurement will be deemed as a reading of ≥ 35 ppb, as per ATS/ERS guidelines for children.(16)
- Spirometry: Spirometry will be classified as obstructed if the FEV1/FVC ratio is less than the Lower Limit of Normal. Obstructed spirometry will undergo a grading of severity based on the pre bronchodilator FEV1 z-score. Post bronchodilator reversibility will be defined as an increase in the highest FEV1 of ≥12% and 200ml from baseline.(17)
  Spirometry parameters used for analysis include:
  - $FEV_1$, in litres, and expressed as percent predicted and z-score;
  - FVC, in litres, in percent predicted and z-score;
  - $FEV_1$/FVC ratio, FEV1/FVC LLN
  - FEF25-75 in litre/second and in percent predicted

Secondary outcomes:

- Assessment of barriers to good control:
  - Current treatment of asthma: set of questions about current medication, reported using descriptive statistics, and percentages
  - Adherence to medication: set of questions about adherence to medication, reported as descriptive statistics and percentages, as well as free text comments.
  - Access to medical care for asthma: set of questions related to access to medical care, reported using descriptive statistics, and percentages
  - Understanding of asthma: set of questions about asthma knowledge, maximum knowledge score is 13, minimum knowledge score is 0, and total range is 13. Higher values represent better knowledge.
  - Illness perception: the Brief Illness Perception questionnaire for asthma. Each of the 8 items in the Brief Illness Perception Questionnaire has a minimum score of 0 and a maximum score of 10. Overall score which represents the degree to which the illness is perceived as threatening or benign.(10,18)
- Asthma-related time off school: set of questions asking about asthma-related time off school, reported as descriptive statistics and percentages
- Assessment of environmental factors:
  - smoking: a set of questions assessing active and passive smoking, reported as descriptive statistics and percentages
  - Environmental factor assessment: a set of questions related to the environment of young people with asthma symptoms, reported using descriptive statistics, and percentages

Correlations with measures of asthma control to other variables, such as adherence to medication, and illness perception will be explored. Cross-analyses and comparison of the data between centres will also be undertaken. A detailed statistical analysis plan is being developed with the assistance of Statistician. Focus group discussions will be transcribed and keywords identified. Qualitative results from the focus groups will in addition inform the direction and acceptability of interventions aimed at removing barriers to poor asthma control.

Transcripts of the focus groups will be analysed using qualitative methods. After familiarizing themselves with the data, a minimum of two researchers will use an inductive coding process, and thematic analysis

For peer review only

will be used to determine patterns in the responses. Themes across data from the various focus group discussions will be identified and coded by each individual researcher manually. Consensus will be reached on the major themes identified using an iterative process. NVivo 11 is used to assist this component of the analysis and to enter transcribed data within codes, categories and themes.

# Intervention development

A preliminary analysis of the data from the survey and focus groups will inform ideas for possible interventions aimed at improving asthma outcomes and understanding around asthma in African children. One component of an intervention that will be assessed is a UK theatre intervention (play) adapted for an African context. The ACACIA study also aims to improve the digital resources available for asthma related services and for children who suffer from asthma. Digital solutions aimed at improved asthma self-management, such as smart peak flow meters and wheeze detectors, which can send information about symptoms to smart phone apps, will be tested by some of the study participants. Transferability of data infrastructure built for ACACIA to other studies or applications will also be explored.

## Theatre intervention development and pilot

The original 'In Control' theatre was written by the Nigerian-born playwright Tunde Euba in collaboration with The Tramshed - (Greenwich and Lewisham Young People's Theatre) GLYPT as an educational performance, aimed at changing attitudes and understanding around asthma. The play revolves around a young protagonist with asthma in a school setting. It is described in a Lancet perspective article. (19) In a UK-based feasibility study 98.7% of 1798 young people in the audience said 'In Control' was enjoyable and 84.6% said that the performance at least somewhat changed how they think or feel about asthma.(20) The theatre will be adapted to the cultural context of sub-Saharan Africa by the original play writer in close collaboration with local arts and theatre groups, and local actors will be trained to perform the play. A pilot study will explore its potential to change understanding of asthma. At each study centre, the pilot study will involve performances in at a minimum of five schools with two performances in each school to audiences of up to 120 children (target age 12 – 16 yrs.), and five additional performances to local community groups, which will be defined after initial community contacts during project recruitment . Outcomes will be measured using short before and after questionnaires about knowledge and attitude towards asthma with additional questions on acceptability in the after-performance questionnaire. Results will be summarised using descriptive statistics and inform any potential future development of a full intervention, if further funding is provided.

# Public and Patient Involvement

Children of the target age group have been involved during the study development, and for example commented on the ACACIA questionnaire and the theatre adaptation. Beyond that, all study sites go through a guided process of developing their Public and Patient Involvement and Engagement (PPIE) activities. PPIE guidelines have been developed centrally in collaboration with the public engagement team at Queen Mary University of London. A PPIE lead at each study site works with the central team to develop a PPIE plan, specific to their local context, and in accordance with the study's aims and objectives. Wherever possible, PPIE activities will be evaluated using established toolkits.

For peer review only - http://bmjopen.bmj.com/site/about/guidelines.xhtml

# ETHICS AND DISSEMINATION

## Ethical review

The ACACIA study has been reviewed by the Queen Mary Ethics of Research Committee in the UK. Each collaborating African centre received ethical approval for the study locally with:

- Malawi: The College of Medicine Research Ethics Committee (COMREC), Reference No: P.10/18/2494, University of Malawi
- Nigeria: Lagos State /Lagos State University Teaching Hospital Health Research and Ethics Committee (NHREC04/04/2008), Reference No: LREC 06/10/1084
- Uganda: Mulago Hospital Research Ethics Committee (MHREC) number MHREC 1514; Institutional Review Board, and National Council for Science and Technology (UNCST) number SS 4940
- South Africa: Biomedical Research Ethics Committee of University of KwaZulu Natal BREC number: BF002/19 and Department of Basic Education KwaZulu Natal and Department of Health KwaZulu Natal
- Zimbabwe: The City Health Ethics Board, Joint Research Ethics Committee (JREC),the Medical Research Council of Zimbabwe (MRCZ); and the Research Council of Zimbabwe (RCZ)
- Ghana: Committee on Human Research, Publication and Ethics (CHRPE) - a joint committee of the School of Medical Science, Kwame Nkrumah University of Science and Technology (KNUST) and the Komfo Anokye Teaching Hospital (KATH), which is registered with the United States Department of Health and Human Services, Office for Human Research Protection (OHRP).

Study direction and progress will be guided by an international ACACIA steering group, chaired by Heather Zar.

## Dissemination

Study results will be published in national and international peer review journals, and at conferences. Scientific results will be published as soon as it is reasonably practical and in compliance with the terms of the funding body. Publication will only be made after QMUL has received written permission from the funding body. Authorship will not necessarily be restricted to individuals named on this protocol; neither is authorship guaranteed to individual named on this protocol. Contributors who do not meet authorship criteria will be listed in ‘Acknowledgements’.
General study information will be displayed on the study’s website, as well as through its twitter account. Furthermore, dissemination of study results in the community is planned as part of PPIE events and activities.

## Data Sharing, Access, and Release

The ACACIA study collects data from children in six African countries. Data will be collected by local research teams in each of the African centres. Participant’s identifiable data will be pseudo-anonymised

For peer review only

before data is analysed or shared with the other centres through a digital platform system. ACACIA will release curated and coded data for open access after the results have been published, within the limits of relevant data protection acts.

## Data protection and confidentiality:

Data collected from participants are stored locally in accordance with local and United Kingdom data protection legislations. The research team will be mindful to implement the highest data protection standards in all countries, especially where little or no local legislation exists. Access to identifiable data will only be possible within each country with local data access restricted to a limited number of reseachers. The ACACIA central platform is hosted behind firewalls within a UK data storage facility. Storage and destruction of data are compliant with the UK Data Protection Act 2018. Only limited potentially identifiable data are contained within the central platform, specifically:

1. Age
2. Ethnicity
3. Gender
4. School (changed into an identifier)
5. Name (changed to an identifier)
6. Exact home location (changed into a square area (Mercator projection, reduced to 2 decimals, resulting in approximately 1km squares))

## Data monitoring

Intermittent random audit of data quality will be performed by members of each local investigating team. Audit by members from the co-ordinating centre in the UK will additionally be performed, including spot checks of entered data, site visits during data collection, and equipment checks.

# ACKNOWLEDGEMENT

The authors would like to thank Professor Farida Fortune from Queen Mary University of London and her technological development team with Andy Carter, and Ian Sempill for their hard work towards developing innovative technological solutions for the study. Further thanks go to Jeremy James, Director of The Tramshed – GLYPT, who collaborates with the study to produce a tailored theatre performance around asthma knowledge and attitudes. The authors would furthermore like to thank all other members of the ACACIA steering group and Co-applicants to the grant for their valuable support of the study, including Professor Chris Griffiths, Professor Robert Walton, and Dr Liz Steed from Queen Mary University of London, as well as Professor Kevin Mortimer from the Liverpool School of Tropical Medicine, and Professor Mike Roberts from University College London Partners.

For peer review only

# REFERENCES


1. Adeloye D, Chan KY, Rudan I et al. An estimate of asthma prevalence in Africa: a systematic analysis. *Croat Med J.* 2013;54:519–31.
2. Global Asthma Network. The Global Asthma Report 2018. Auckland, New Zealand. 2018. http://globalasthmareport.org/Global Asthma Report 2018.pdf (accessed 29th October 2019).
3. Id OBO, Aderibigbe SA, Ayuk AC, Desalu OO, Oridota OE, Olufemi O, et al. The prevalence of asthma and allergic rhinitis in Nigeria: A nationwide survey among children, adolescents and adults. *Plos One* September 13 https://doi.org/10.1371/journal.pone.0222281 (accessed 29th October 2019).
4. Rylance S, Nightingale R, Naunje A et al. Lung health and exposure to air pollution in Malawian children (CAPS): a cross-sectional study. *Thorax* 2019;74:1070–7.
5. Iraqi B, Mahraoui C. Evaluation of asthma control among children in the Maghreb and sub-SaharIan Africa. *Eur Respir J.* 2016;48(suppl 60).
6. Shimwela M, Mwita JC, Mwandri M et al. Asthma prevalence, knownledge and perceptions among secondary school pupils in rural and urban coastal districts in Tanzania. *BMC Public Health* 2014;14:387.
7. Harris K, Mosler G, Williams SA, et al. Asthma control in London secondary school children. *J Asthma* 2017;54(10):1033–40.
8. Harris K, Mosler G, Grigg J. Intervention to improve adolescents' asthma control: a cluster randomised controlled trial protocol. *BMJ Open* 2019;9(4):e025867.
9. Global Asthma Network. Global Study Instruments for 13/14 year olds. http://www.globalasthmanetwork.org/surveillance/manual/study13.php (accessed 29th OCtober 2019).
10. Broadbent E, Petrie KJ, Main J et al. The Brief Illness Perception Questionnaire. *J Psychosom Res* 2006;60(6):631–7.
11. Liverpool School of Tropical Medicine. February 2018, IMPALA/ LuLi questionnaires https://www.lstmed.ac.uk/research/centres-and-units/impala/impala-outputs-resources (accessed 29th October 2019).
12. Schatz M, Sorkness CA, Li JT et al. Asthma Control Test: Reliability, validity, and responsiveness in patients not previously followed by asthma specialists. *J Allergy Clin Immunol* 2006;117(3):549–56.
13. Global Initiative for Asthma. Pocket Guide for Asthma Management and Prevention: GINA executive summary. 2018. 28 p.
14. Quanjer PH, Stanojevic S, Cole TJ et al. Multi-ethnic reference values for spirometry for the 3–95-yr age range: the global lung function 2012 equations. *Eur Respir J* 2012;40(6):1324–43.
15. Reddel HK, Taylor DR, Bateman ED et al. An official American Thoracic Society/European Respiratory Society statement: asthma control and exacerbations: standardizing endpoints for clinical asthma trials and clinical practice. *Am J Respir Crit Care Med* 2009 Jul 1;180(1):59-99.
16. Dweik RA, Boggs PB, Erzurum SC et al. An Official ATS Clinical Practice Guideline: Interpretation of Exhaled Nitric Oxide Levels (FENO) for Clinical Applications. *Am Thorac Soc Doc* 2011;184:602–15.
17. Swanney MP, Ruppel G, Enright PL et al. Using the lower limit of normal for the FEV 1/ FVC ratio reduces the misclassification of airway obstruction. *Thorax* 2008;63:1046–51.
18. Broadbent E, Wilkes C, Koschwanez H, et al. A systematic review and meta-analysis of the Brief Illness Perception Questionnaire. *Psychol Heal* 2015;30(11):1361–85.
19. Mosler G, Euba T. Taking control through drama. *Lancet* 2017;6736(17):1–2.


For peer review only

20. Mosler G, Harris K, Grigg J. 'In Control' - changing asthma attitudes in adolescents through drama. *Eur Respir J* 2018 52: PA4604.

# AUTHORS CONTRIBUTIONS

GM is the main author of this manuscript and the Central Coordinator for ACACIA. JG is the Director of the ACACIA study providing overall direction and supervision. JG has contributed directly to this manuscript by proof-reading and commenting on its content. VO is the UK-based PhD student and Respiratory Physiologist of the study. She contributed to the manuscript through proof-reading and editing. All Principal Investigators (PIs) of each ACACIA centre (EA-Y, OA, RM, HAM, RN, SR, IT) have furthermore read and commented on the manuscript's content.

# FUNDING STATEMENT

This research was funded by the National Institute for Health Research (NIHR) (project reference) using UK aid from the UK Government to support global health research. The views expressed in this publication are those of the author(s) and not necessarily those of the NIHR or the UK Department of Health and Social Care (17/63/38).

# COMPETING INTEREST STATEMENTS

JG reports personal fees from GSK, personal fees from Vifor Pharmaceuticals, personal fees from Novartis, personal fees from BV Pharma, personal fees from AstraZeneca, outside the submitted work.

# LIST OF FIGURES

Figure 1: Participant Flow Chart

For peer review only

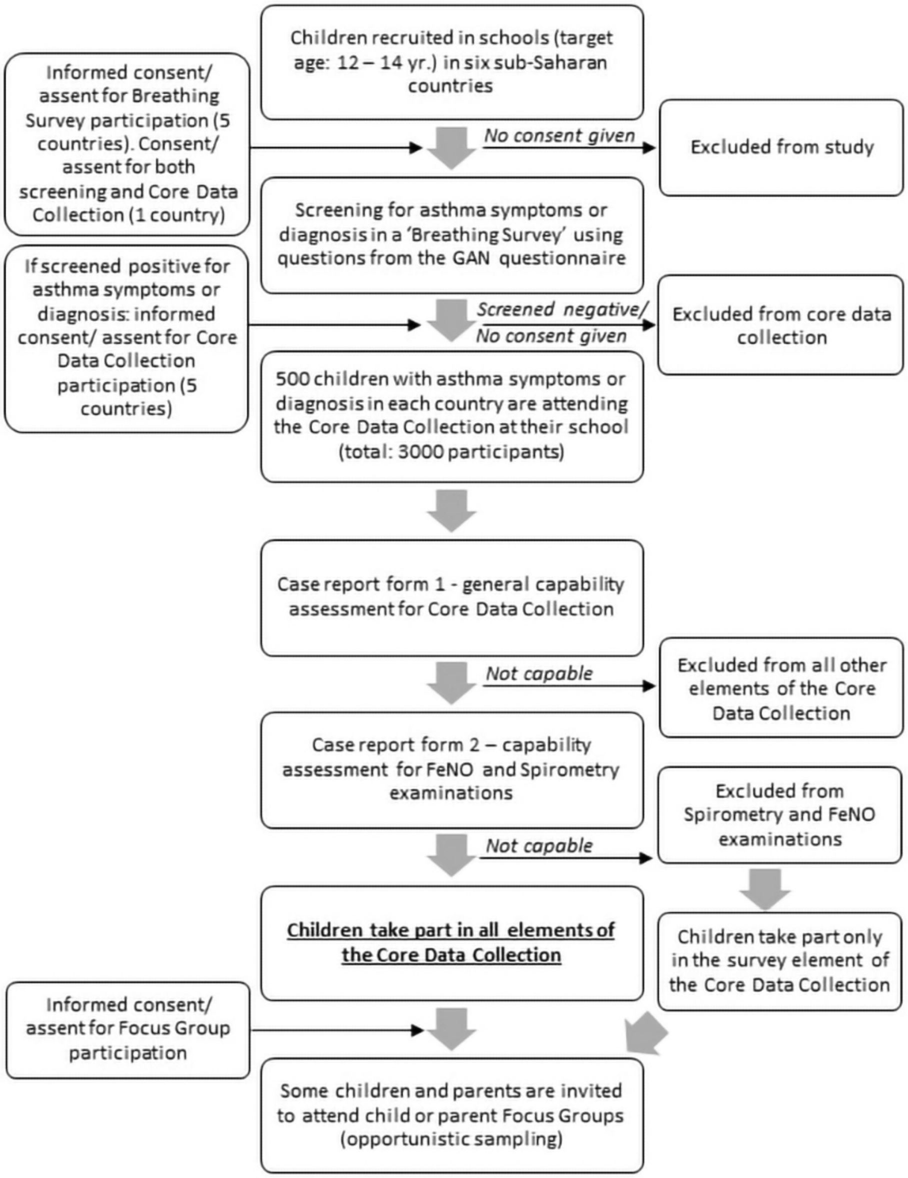


Participant Flow Chart

75x95mm (300 x 300 DPI)

# Supplementary File
# ACACIA Questionnaire - text only version

Participants will fill in a digital version of this questionnaire. They will be closely supported by facilitators throughout. Pictures are included in the digital questionnaire for all medication, including spacers, as well as cookers/main stoves, and light sources. In the digital version questions are automatically skipped, if the condition for the question is not fulfilled, or if they have answered the question at an earlier stage in the survey (e.g. as part of a conditional question).

---

## Section 1: Personal details

1. **What is your name? ______________________________________________________**

2. **Date of birth (DD/MM/YYYY):** ***(digital: drop down menu)***

3. **Please find your home location on the map with the help of the visiting researcher.**

4. **Are you male or female?**
   - ○ Male
   - ○ Female

5. **Which school class or school year are you in? ____________**

6. **How would you describe your ethnicity?** ***(digital: drop down menu with follow up ' please specify for 'mixed race' and 'other')***
   - ○ Black
   - ○ White
   - ○ East Asian, such as Chinese, Japanese, or Korean
   - ○ South Asian, such as Indian, or Bangladeshi
   - ○ Mixed race, please specify: ______________________
   - ○ Other ethnicity, please specify: __________________________

7. **Has a doctor, pharmacist, nurse, or healthcare worker ever said that you suffer from asthma?**
   - ○ Yes
   - ○ No

For peer review only



*The questionnaire was based on work from the School-based Asthma Project, funded by the NIHR (National Institute for Health Research) CLAHRC (Collaboration for Leadership in Applied Health Research and Care) North Thames. The license for the Asthma Control Test (ACT) was donated to the ACACIA study through the NIHR CLAHRC North Thames as part of the initial work.*

For peer review only - http://bmjopen.bmj.com/site/about/guidelines.xhtml

- Don’t know

**8. Has a doctor, pharmacist, nurse, or healthcare worker ever said that you suffer from any of the following conditions?** (select all that apply)

- Atopic dermatitis, also sometimes known as eczema
- Hay fever and, or pollen allergy.
- Other allergies**, please specify:** ____________________________________
- Don’t know

**9. Have you been treated for Tuberculosis/TB?**

- Yes
- No
- I’d rather not say
- Don’t know

**10. Does one or both of your parents suffer from any of the following conditions:**

- Asthma
- Allergies
- Atopic dermatitis, also sometimes known as eczema
- None of the above
- Don’t know

For peer review only



*The questionnaire was based on work from the School-based Asthma Project, funded by the NIHR (National Institute for Health Research) CLAHRC (Collaboration for Leadership in Applied Health Research and Care) North Thames. The license for the Asthma Control Test (ACT) was donated to the ACACIA study through the NIHR CLAHRC North Thames as part of the initial work.*

For peer review only - http://bmjopen.bmj.com/site/about/guidelines.xhtml

# Section 2: Asthma Control Test (ACT) and GINA questions

## ACT

1. **In the past 4 weeks, how much of the time did your asthma keep you from getting as much done at work, school or home?**

- ◯ All of the time
- ◯ Most of the time
- ◯ Some of the time
- ◯ A little of the time
- ◯ None of the time

2. **In the past 4 weeks, how often have you had shortness of breath?**

- ◯ More than once a day
- ◯ Once a day
- ◯ 3 to 6 times a week
- ◯ Once or twice a week
- ◯ Not at all

3. **In the past 4 weeks, how often did your asthma symptoms (wheezing, coughing, chest tightness, shortness of breath) wake you up at night or earlier than usual in the morning?**

- ◯ 4 or more nights a week
- ◯ 2 to 3 nights a week
- ◯ Once a week
- ◯ Once or twice (in 4 weeks)
- ◯ Not at all

***If you have a reliever or rescue inhaler***

4. **In the past 4 weeks, how often have you used your rescue/reliever inhaler?**

- ◯ 3 or more times per day
- ◯ Once or twice per day
- ◯ 2 or 3 times per week
- ◯ Once a week or less
- ◯ Not at all



*The questionnaire was based on work from the School-based Asthma Project, funded by the NIHR (National Institute for Health Research) CLAHRC (Collaboration for Leadership in Applied Health Research and Care) North Thames. The license for the Asthma Control Test (ACT) was donated to the ACACIA study through the NIHR CLAHRC North Thames as part of the initial work.*

For peer review only - http://bmjopen.bmj.com/site/about/guidelines.xhtml
For peer review only

**5. How would you rate your asthma control during the last 4 weeks?**

- Not controlled at all
- Poorly controlled
- Somewhat controlled
- Well controlled
- Completely controlled

# GINA

**1. In the past 4 weeks, have you had asthma symptoms during the day more than twice a week?**

- Yes
- No

**2. In the past 4 weeks, did you have any night waking due to asthma?**

- Yes
- No

***If you have a reliever or rescue inhaler***

**3. In the past 4 weeks, did you need your reliever more than twice/week?**

- Yes
- No

**4. In the past 4 weeks, did you have any activity limitation due to asthma?**

- Yes
- No

For peer review only



*The questionnaire was based on work from the School-based Asthma Project, funded by the NIHR (National Institute for Health Research) CLAHRC (Collaboration for Leadership in Applied Health Research and Care) North Thames. The license for the Asthma Control Test (ACT) was donated to the ACACIA study through the NIHR CLAHRC North Thames as part of the initial work.*

For peer review only - http://bmjopen.bmj.com/site/about/guidelines.xhtml

# Section 3: Medicines and adherence

**1. Do you take any medications for your asthma or wheeze?**

○ Yes

○ No

***If yes,***

**2. What type of medications do you usually use for your wheezing or asthma?** ***(pictures available in digital version)***

| | | | | | | |
|---|---|---|---|---|---|---|
| **Blue inhaler**<br>Subumol, Salbutamol, Ventolin<br>☐ | **Yellow inhaler**<br>Albuterol, Proventil<br>☐ | **Green inhaler**<br>Salmeterol Serevent<br>☐ | **Bricanyl**<br>- Terbutaline DPI<br>☐ | **Combivent HFA**<br>Salbutamol Ipratropium<br>☐ | **Duovent**<br>Feneterol Ipratropium<br>☐ | **Levosalbutamol Syrup**<br>Levolin<br>☐ |

| | | | | | | |
|---|---|---|---|---|---|---|
| **Ventolin tablets**<br>Salbutamol<br>☐ | **Ascoril Syrup**<br>☐ | **Salbutamol syrup**<br>☐ | **Brown inhaler**<br>Becasone, Beclometasone, Becotide<br>☐ | **Red inhaler**<br>Ciclesonide, Alvesco<br>☐ | **Purple inhaler**<br>Fluticasone/ Salmeterol Seretide<br>☐ | **Red/white inhaler**<br>Budesonide/ Formoterol Symbicort<br>☐ |

| | | | | | | |
|---|---|---|---|---|---|---|
| **Brown/white inhaler**<br>Budesonide Pulmicort<br>☐ | **Orange inhaler**<br>Fluticasone Flixotide<br>☐ | **Flixotide accuhaler DPI**<br>☐ | **Seretide Accuhaler**<br>☐ | **Asthmanex twisthaler**<br>☐ | **Ciclovent, generic of Ciclesonide**<br>☐ | **Steroid tablets**: Prednisolone<br>☐ |

| | | | | | | |
|---|---|---|---|---|---|---|
| **Theophylline tablets** or Nuelin SA<br>☐ | **Ventolin tablets**<br>Salbutamol<br>☐ | **Franol, Asthmanol**<br>Ephedrine theophylline<br>☐ | **LTRA, Montelukast tablets**<br>Topraz, Singulair, Sintrine, Monte-Air<br>☐ | **Cough syrup**<br>☐ | **Comple-mentary, herbal, or alternative medicines**<br>☐ | **Nebulizer**<br>☐ |

☐ **I have other medicine,** Please name: ____________________________________________

For peer review only



*The questionnaire was based on work from the School-based Asthma Project, funded by the NIHR (National Institute for Health Research) CLAHRC (Collaboration for Leadership in Applied Health Research and Care) North Thames. The license for the Asthma Control Test (ACT) was donated to the ACACIA study through the NIHR CLAHRC North Thames as part of the initial work.*

For peer review only - http://bmjopen.bmj.com/site/about/guidelines.xhtml

***If you are using any MDI (puffer) inhaler:***

**3. Do you use a spacer for your inhaler at least some of the time**

- Yes
- No
- Don't know

***If you are using a spacer:***

**4. How often do you use your spacer?**

- With each dose of medication
- Some of the time
- Never

***If you are using a spacer:***

**5. Which type of spacer are you usually using?**

- Home-made bottle spacer
- Bought plastic spacer

***If you are taking reliever medicine when your symptoms get worse (for example blue or yellow reliever inhalers)***

**6. Do you sometimes not use your reliever medicine when you would need it? *(digital: look up button with list of reliever medicine)***

- All of the time
- Some of the time
- None of the time
- I do not have any reliever medicine

***If you sometimes forget your reliever medicine,***

7. **Would you like to tell us why?** ______________________________________________

______________________________________________________________________

***If you have regular preventer medicine, usually taken once or twice every day (for example a preventer inhaler)***

**8. Do you sometimes not take your regular daily preventer medicine? *(digital: look up button with list of preventer/controller medicine)***

- All of the time
- Some of the time
- None of the time
- I do not have any preventer medicine


*The questionnaire was based on work from the School-based Asthma Project, funded by the NIHR (National Institute for Health Research) CLAHRC (Collaboration for Leadership in Applied Health Research and Care) North Thames. The license for the Asthma Control Test (ACT) was donated to the ACACIA study through the NIHR CLAHRC North Thames as part of the initial work.*
For peer review only - http://bmjopen.bmj.com/site/about/guidelines.xhtml
For peer review only

***If you sometimes forget to take your preventer medicine,***

**9. Would you like to tell us why?**

________________________________________________________________________________

________________________________________________________________________________

For peer review only



*The questionnaire was based on work from the School-based Asthma Project, funded by the NIHR (National Institute for Health Research) CLAHRC (Collaboration for Leadership in Applied Health Research and Care) North Thames. The license for the Asthma Control Test (ACT) was donated to the ACACIA study through the NIHR CLAHRC North Thames as part of the initial work.*

For peer review only - http://bmjopen.bmj.com/site/about/guidelines.xhtml

# Section 4 Medical attention

***If you are taking medicine (section 3)***

**1. How do you usually get your medicine for asthma or wheezing?**

- Prescribed by a nurse, or a doctor at a clinic or hospital
- Bought directly from a pharmacy without prescription
- From a traditional healer or herbalist
- I see different people about my medicines
- My parent or carer gets the medicine for me, I usually don't come along
- Other: ___________________________
- Don't know

***If prescribed by a nurse, or a doctor or bought from a pharmacist***

**2. When you see your doctor, nurse or pharmacist about your medicine, would they usually ask you questions about your asthma or wheezing?**

- Yes
- Sometimes
- They only talk to my parent or carer
- No, they don't talk to me or my parents/carers about my asthma or wheezing
- Don't know

**3. Where do you usually get medical help when your asthma or wheezing gets suddenly worse? Tick all that apply.**

- Hospital
- Emergency room
- Local clinic or health centre
- Pharmacy
- Traditional healer, traditional doctor or herbalist
- Other: ___________________________
- Don't know

**4. In the past year, how often did you suddenly need to see a doctor or nurse at a hospital, emergency room, or clinic because of your asthma or wheezing?**

- 4 or more times
- 2-3 times
- Once
- Not at all
- Don't know



*The questionnaire was based on work from the School-based Asthma Project, funded by the NIHR (National Institute for Health Research) CLAHRC (Collaboration for Leadership in Applied Health Research and Care) North Thames. The license for the Asthma Control Test (ACT) was donated to the ACACIA study through the NIHR CLAHRC North Thames as part of the initial work.*

For peer review only - http://bmjopen.bmj.com/site/about/guidelines.xhtml

5. **In the past year, how many times would you have liked to get medical help because of your wheezing or asthma, but were not able to do so?**
   - ○ 4 or more times
   - ○ 2-3 times
   - ○ Once
   - ○ Not at all
   - ○ Don't know

   ***If at least once,***
   **Would you like to tell us why?** ______________________________________________

For peer review only



*The questionnaire was based on work from the School-based Asthma Project, funded by the NIHR (National Institute for Health Research) CLAHRC (Collaboration for Leadership in Applied Health Research and Care) North Thames. The license for the Asthma Control Test (ACT) was donated to the ACACIA study through the NIHR CLAHRC North Thames as part of the initial work.*

For peer review only - http://bmjopen.bmj.com/site/about/guidelines.xhtml

# Section 5: School activity

### *(if diagnosis of asthma)*

1. **How many times have you missed at least part of a school day due to your asthma in the past four weeks?**

- 4 or more times
- 2-3 times
- Once
- Not at all

2. **Do you feel that your asthma has a bad impact on how well you do in any of your classes or exams?**

- My asthma doesn't have an impact at all
- My asthma has some impact
- My asthma has a big impact

3. **Have you ever been teased, made fun of or bullied because of your asthma?**

- A lot
- A little
- Never
- I'd rather not say

For peer review only



*The questionnaire was based on work from the School-based Asthma Project, funded by the NIHR (National Institute for Health Research) CLAHRC (Collaboration for Leadership in Applied Health Research and Care) North Thames. The license for the Asthma Control Test (ACT) was donated to the ACACIA study through the NIHR CLAHRC North Thames as part of the initial work.*

For peer review only - http://bmjopen.bmj.com/site/about/guidelines.xhtml

# Section 6: Brief-Illness Perception (BIP) Questionnaire and peer support

*(if diagnosis of asthma)*

## BIP

1. **How much does your asthma affect your life?**

| 0 | 1 | 2 | 3 | 4 | 5 | 6 | 7 | 8 | 9 | 10 |
|---|---|---|---|---|---|---|---|---|---|---|
| No affect at all | | | | | | | | | | Severely affects my life |

2. **How long do you think your asthma will continue?**

| 0 | 1 | 2 | 3 | 4 | 5 | 6 | 7 | 8 | 9 | 10 |
|---|---|---|---|---|---|---|---|---|---|---|
| A very short time | | | | | | | | | | Forever |

3. **How much control do you feel you have over your asthma?**

| 0 | 1 | 2 | 3 | 4 | 5 | 6 | 7 | 8 | 9 | 10 |
|---|---|---|---|---|---|---|---|---|---|---|
| Absolutely no control | | | | | | | | | | Extreme amount of control |

4. **How much do you think your treatment can help your asthma?**

| 0 | 1 | 2 | 3 | 4 | 5 | 6 | 7 | 8 | 9 | 10 |
|---|---|---|---|---|---|---|---|---|---|---|
| Not at all | | | | | | | | | | Extremely helpful |

5. **How much do you experience symptoms from your asthma?**

| 0 | 1 | 2 | 3 | 4 | 5 | 6 | 7 | 8 | 9 | 10 |
|---|---|---|---|---|---|---|---|---|---|---|
| No symptoms at all | | | | | | | | | | Many severe symptoms |

6. **How concerned are you about your asthma?**

| 0 | 1 | 2 | 3 | 4 | 5 | 6 | 7 | 8 | 9 | 10 |
|---|---|---|---|---|---|---|---|---|---|---|
| Not at all concerned | | | | | | | | | | Extremely concerned |

7. **How well do you feel you understand your asthma?**

| 0 | 1 | 2 | 3 | 4 | 5 | 6 | 7 | 8 | 9 | 10 |
|---|---|---|---|---|---|---|---|---|---|---|
| Don't understand at all | | | | | | | | | | Understand very clearly |

8. **How much does your asthma affect you emotionally (e.g. does it make you angry, scared, upset or depressed)?**

| 0 | 1 | 2 | 3 | 4 | 5 | 6 | 7 | 8 | 9 | 10 |
|---|---|---|---|---|---|---|---|---|---|---|
| Not at all affected emotionally | | | | | | | | | | Extremely affected emotionally |



*The questionnaire was based on work from the School-based Asthma Project, funded by the NIHR (National Institute for Health Research) CLAHRC (Collaboration for Leadership in Applied Health Research and Care) North Thames. The license for the Asthma Control Test (ACT) was donated to the ACACIA study through the NIHR CLAHRC North Thames as part of the initial work.*

For peer review only - http://bmjopen.bmj.com/site/about/guidelines.xhtml

# PEER SUPPORT

**1. How important do you think peer support (from colleagues and friends) is for people with asthma?**

| 0 | 1 | 2 | 3 | 4 | 5 | 6 | 7 | 8 | 9 | 10 |
|---|---|---|---|---|---|---|---|---|---|---|
| Not at all important | | | | | | | | | | Extremely important |

For peer review only



The questionnaire was based on work from the School-based Asthma Project, funded by the NIHR (National Institute for Health Research) CLAHRC (Collaboration for Leadership in Applied Health Research and Care) North Thames. The license for the Asthma Control Test (ACT) was donated to the ACACIA study through the NIHR CLAHRC North Thames as part of the initial work.

For peer review only - http://bmjopen.bmj.com/site/about/guidelines.xhtml

# Section 7: Understanding about asthma

**In this questionnaire we ask some questions about reliever and preventer inhalers. Ask the researchers with you to show you some examples.**

| | | | |
|---|---|---|---|
| 1. Your asthma would get worse if people smoke around you | Don't know | Agree | Disagree |
| 2. Preventer inhalers only work if you feel them working immediately | Don't know | Agree | Disagree |
| 3. Asthma is well controlled if you need to take a reliever inhaler once a day | Don't know | Agree | Disagree |
| 4. Using a spacer with an asthma inhaler makes it easier to get the medicine into the lungs | Don't know | Agree | Disagree |
| 5. A tight feeling in your chest can be a symptom for asthma | Don't know | Agree | Disagree |
| 6. You can get addicted to asthma inhalers | Don't know | Agree | Disagree |
| 7. Both stress and cold air can be triggers for asthma symptoms | Don't know | Agree | Disagree |
| 8. People with asthma are less likely to get swollen airways | Don't know | Agree | Disagree |
| 9. You should get a person straight to hospital if they cannot talk or walk anymore during an asthma attack | Don't know | Agree | Disagree |
| 10. A spacer should only be used by small children | Don't know | Agree | Disagree |
| 11. The reliever inhaler prevents the airways from becoming swollen | Don't know | Agree | Disagree |
| 12. In an asthma emergency, you can give the reliever inhaler 10 puffs in a row | Don't know | Agree | Disagree |
| 13. People with asthma should take a preventer inhaler when they have an asthma attack | Don't know | Agree | Disagree |
| 14. Several young people die from asthma every year in my country | Don't know | Agree | Disagree |
| 15. Young people sometimes make fun of asthma | Don't know | Agree | Disagree |

For peer review only



*The questionnaire was based on work from the School-based Asthma Project, funded by the NIHR (National Institute for Health Research) CLAHRC (Collaboration for Leadership in Applied Health Research and Care) North Thames. The license for the Asthma Control Test (ACT) was donated to the ACACIA study through the NIHR CLAHRC North Thames as part of the initial work.*

For peer review only - http://bmjopen.bmj.com/site/about/guidelines.xhtml

# Section 8: Smoking, Home living, and Exposure

## SMOKING

1. **Have you ever smoked cigarettes or tobacco?**
   - ◌ Yes
   - ◌ No
   - ◌ I'd rather not say

   ***If yes:***
2. **How often do you smoke cigarettes or tobacco?**
   - ◌ Every day
   - ◌ 5-6 days a week
   - ◌ 3-4 days a week
   - ◌ 1-2 days a week
   - ◌ Less than once a week

3. **Does anyone smoke cigarettes or tobacco inside your home at least once a week?**
   - ◌ Yes
   - ◌ No
   - ◌ I'd rather not say

## EXPOSURE

| | | | |
|---|---|---|---|
| **4. Is the main cooker in your home usually used outside?** | **Don't know** | **Yes** | **No** |
| **5. Is the main cooker in your home electric?** | **Don't know** | **Yes** | **No** |
| **6. Does the road nearest your home have cars, buses, or trucks driving along it all the time?** | **Don't know** | **Yes** | **No** |
| **7. Do you have any animals or pets living with you at home?** | **Don't know** | **Yes** | **No** |
| **8. Do you live near a dump site where they burn waste at least once a month?** | **Don't know** | **Yes** | **No** |
| **9. Do you breathe in smoke from bush fires at least once a month?** | **Don't know** | **Yes** | **No** |
| **10. Does anyone living at your home use cleaning spray?** | **Don't know** | **Yes** | **No** |
| **11. Does anyone living at your home use insecticide spray?** | **Don't know** | **Yes** | **No** |



*The questionnaire was based on work from the School-based Asthma Project, funded by the NIHR (National Institute for Health Research) CLAHRC (Collaboration for Leadership in Applied Health Research and Care) North Thames. The license for the Asthma Control Test (ACT) was donated to the ACACIA study through the NIHR CLAHRC North Thames as part of the initial work.*

For peer review only - http://bmjopen.bmj.com/site/about/guidelines.xhtml

| | | | |
|---|---|---|---|
| 12. Does anyone living at your home use deodorant spray? | Don't know | Yes | No |
| 13. Does anyone at your home burn mosquito coils? | Don't know | Yes | No |
| 14. Does anyone at your home burn incense? | Don't know | Yes | No |
| 15. Is your home sometimes air conditioned? | Don't know | Yes | No |
| 16. Do you usually use electric lights at your house? | Don't know | Yes | No |

***If the main cooker in your home IS NOT electric***

**17. What type of cooker is the main stove at your home? Tick all that apply.**

- ☐ Gas cooker, or mixed gas and electric
- ☐ Open fire or three stone stove
- ☐ Cooker using coal, wood, or other solid fuel
- ☐ Cooker using kerosene, oil, or another liquid
- ☐ Solar cooker
- ☐ Others: ____________________
- ☐ Don't know

***If you DO NOT usually use electric lights,***

**18. What sort of lighting do you use? Tick all that apply.**

- ☐ **Candle (wax)**
- ☐ **Open fire**
- ☐ **Kerosene lamp**
- ☐ **Oil wick lamp**
- ☐ **Solar lantern**
- ☐ **Torch**
- ☐ **Rechargeable/ battery lamp**
- ☐ **Battery powered inverter**

For peer review only



*The questionnaire was based on work from the School-based Asthma Project, funded by the NIHR (National Institute for Health Research) CLAHRC (Collaboration for Leadership in Applied Health Research and Care) North Thames. The license for the Asthma Control Test (ACT) was donated to the ACACIA study through the NIHR CLAHRC North Thames as part of the initial work.*

For peer review only - http://bmjopen.bmj.com/site/about/guidelines.xhtml

| | |
|---|---|
| ☐ | Gasoline/diesel powered generator |
| ☐ | Crop residue |
| ☐ | Others: ______________________________________________ |
| ☐ | Don't know |

# Researcher Section 9

***to be filled in by the researcher***

1. **Date of questionnaire session (DD/MM/YYYY): ______________________**
2. **School: _______________________________________________**
3. **Section 1, Question 3: Lat:_____________, Long: ____________**
4. **What kind of area is the home in?**
   - ○ City or town
   - ○ Suburban
   - ○ Village or countryside
2. **Distance from home to nearest major road (map)**
   - ○ Less than 50m
   - ○ 50m-100m
   - ○ 100m-250m
   - ○ 250m-1000m
   - ○ More than 1000m
3. **Distance from school to nearest major road (measured on map)**
   - ○ Less than 50m
   - ○ 50m – 100m
   - ○ 100m - 250m
   - ○ 250m – 1000m
   - ○ More than 1000m
4. **School yard surface**
   - ○ Loose dirt
   - ○ Packed dirt
   - ○ Paved broken
   - ○ Paved

   Other: _______________________________________


*The questionnaire was based on work from the School-based Asthma Project, funded by the NIHR (National Institute for Health Research) CLAHRC (Collaboration for Leadership in Applied Health Research and Care) North Thames. The license for the Asthma Control Test (ACT) was donated to the ACACIA study through the NIHR CLAHRC North Thames as part of the initial work.*
For peer review only - http://bmjopen.bmj.com/site/about/guidelines.xhtml
For peer review only
